# High climate model dependency of Pliocene Antarctic ice-sheet predictions

**DOI:** 10.1038/s41467-018-05179-4

**Published:** 2018-07-18

**Authors:** Aisling M. Dolan, Bas de Boer, Jorge Bernales, Daniel J. Hill, Alan M. Haywood

**Affiliations:** 1 School of Earth and Environment, University of Leeds, Leeds, LS2 9JT UK; 2 Institute for Marine and Atmospheric Research Utrecht, Utrecht University, Utrecht, 3584 CC The Netherlands; 3 Helmholtz Centre Potsdam, GFZ German Research Centre for Geosciences, Potsdam, 14473 Germany; 4 MARUM Centre for Marine Environmental Sciences, University of Bremen, Bremen, 28359 Germany

## Abstract

The mid-Pliocene warm period provides a natural laboratory to investigate the long-term response of the Earth’s ice-sheets and sea level in a warmer-than-present-day world. Proxy data suggest that during the warm Pliocene, portions of the Antarctic ice-sheets, including West Antarctica could have been lost. Ice-sheet modelling forced by Pliocene climate model outputs is an essential way to improve our understanding of ice-sheets during the Pliocene. However, uncertainty exists regarding the degree to which results are model-dependent. Using climatological forcing from an international climate modelling intercomparison project, we demonstrate the high dependency of Antarctic ice-sheet volume predictions on the climate model-based forcing used. In addition, the collapse of the vulnerable marine basins of Antarctica is dependent on the ice-sheet model used. These results demonstrate that great caution is required in order to avoid making unsound statements about the nature of the Pliocene Antarctic ice-sheet based on model results that do not account for structural uncertainty in both the climate and ice sheet models.

## Abstract (web summary)

Ice sheet models forced by climate model output indicate ice-sheet retreat during the Pliocene, yet concerns remain regarding potential model bias. Here, the authors present results from the Pliocene Ice-sheet Modelling Intercomparison Project, and show that results are highly dependent on the model forcing used.

## Introduction

A warm interval during the Late Pliocene (the mid-Piacenzian Warm Period; mPWP; 3.264–3.025 Ma) offers the most recent time in Earth history where global annual mean temperatures were on average 1.8–3.6 °C higher^1^ and while variable, carbon dioxide concentrations are suggested to be elevated relative to the pre-industrial^2,3^. Estimates of sea level change between 10 and 30 m above present day^4–8^ have been reconstructed based on geological evidence, suggesting that a significant contribution to sea level from both the Greenland and Antarctic Ice-sheets (AIS) is required at certain intervals within the mPWP. This makes the mPWP an important interval to understand in terms of future climate change and ice-sheet stability^9^.

The extent and nature of the AIS during the mPWP remains uncertain. Sedimentological evidence from the ANDRILL core suggests that there were periods where no ice was present on West Antarctica (WAIS) during the Pliocene^10^. Marine records from the margin of East Antarctica also have been interpreted to suggest a dynamic EAIS margin at this time^11–13^. A more recent study used a combination of cosmogenic nuclide exposure ages alongside ice-sheet modelling to suggest that the EAIS continental interior could have been up to 600 m higher than present^14^. Such limited direct proxy evidence means that ice-sheet modelling is particularly important in understanding more about the behaviour and dynamics of ice-sheets during the Pliocene.

Previous modelling studies have supported the geological inferences and shown the potential for significant ice-sheet collapse events during the Pliocene in West Antarctica^15^. However, models are not in agreement as to what degree the EAIS could vary during the warmer intervals of the Pliocene^16^. Transient simulations with ice-sheet models (ISMs) have not shown significant ice retreat in the areas that proxy data suggests^17,18^. However, studies using climate forcings derived from climate models with imposed Pliocene boundary conditions (e.g. the PRISM boundary conditions; Pliocene Research, Interpretation and Synoptic Mapping) to force shallow ice approximation (SIA) ISMs have shown the potential for ice-sheet reduction^19,20^. More recent studies have advocated the inclusion of alternative physics within ISMs in order to facilitate the simulation of different extents of the Pliocene AIS^21–23^, and these have shown the potential for marine incursions into the Wilkes and Aurora subglacial basins (SGB). Nevertheless, it is difficult to perform a true comparison between such previous studies due to the very different models and modelling frameworks that have been adopted.

The Pliocene Model Intercomparison Project (Part 1; PlioMIP1)^1,24^ offers a unique opportunity to test the dependency of ice-sheet simulations to the climate model forcing that is implemented. The models used in PlioMIP1 are similar in terms of complexity (all having atmosphere, land-surface, coupled ocean and sea-ice components) and are all models that were represented in the IPCC 5th Assessment report^25^. All of the PlioMIP1 models implemented PRISM3 boundary conditions for their mid-Pliocene experiment, which included a reduced AIS^26^ (see Methods). It has been shown previously, that whilst the PlioMIP1 model results have commonalities in terms of the large-scale features of Pliocene climate, there is substantial regional variation in the sensitivity of models to the implementation of the Pliocene boundary conditions^1^. Therefore, it is useful to assess the impact of any simulated regional climate differences over Antarctica on the prediction of the Pliocene.

Here, we present results from the Pliocene Ice-sheet Modelling Intercomparison Project (PLISMIP)^27^, which was initiated in order to understand the extent to which using different climate and ISMs changes the predicted ice-sheet configuration for the warm Pliocene. We use output from the climate modelling intercomparison project, PlioMIP^1,24^, to force ice-sheet simulations of the mPWP. The significance of using different ISMs has been demonstrated previously^28^ and therefore, here we force three different ISMs (representing different levels of complexity; ANICE^18^, SICOPOLIS^29,30^ and BASISM^31^) and simulate equilibrium ice-sheets following the initial PLISMIP experimental set-up^27^ (see Methods). Using our ensemble of simulations, we demonstrate that there is a high level of climate model dependency in the reconstruction of the present-day AIS as well as the mPWP AIS. This is especially so in terms of the reconstructed ice volume. For the Pliocene, we use both PRISM3 and present-day AIS configurations to initialise our ISMs. We show that in general when using PRISM3 initial conditions in the ISMs, there is a consistent reduction in the broad extent of the WAIS in the Pliocene. However, some ensemble members retain ice over the West Antarctic Islands, while others lose this ice completely. When using present-day ice-sheet initial conditions different outcomes arise. One model predicts a WAIS with an extent equivalent to modern, whereas the remaining ice-sheet simulations reduce the size of WAIS. Over East Antarctica, the areas of ice reduction in the Pliocene (when compared to modern) differ according to which climate forcing but also importantly which of the ISMs is employed. It is worthy of note that there is more consistency in the results when present-day initial conditions are used within the ISMs. We conclude that it is very important to acknowledge the likely biases in Pliocene ice-sheet simulation based on a single model or even combination of models. Whilst it is useful to show the plausible range in Pliocene AIS reconstructions as a result of choosing different GCMs or ISMs, it is difficult to use the results presented here to directly inform us about the exact configuration of the AIS during the warmest intervals of the Pliocene due to insufficient geological data constraints on the ice-sheet itself.

## Results

### Pliocene climate over Antarctica

**Fig. 1:**
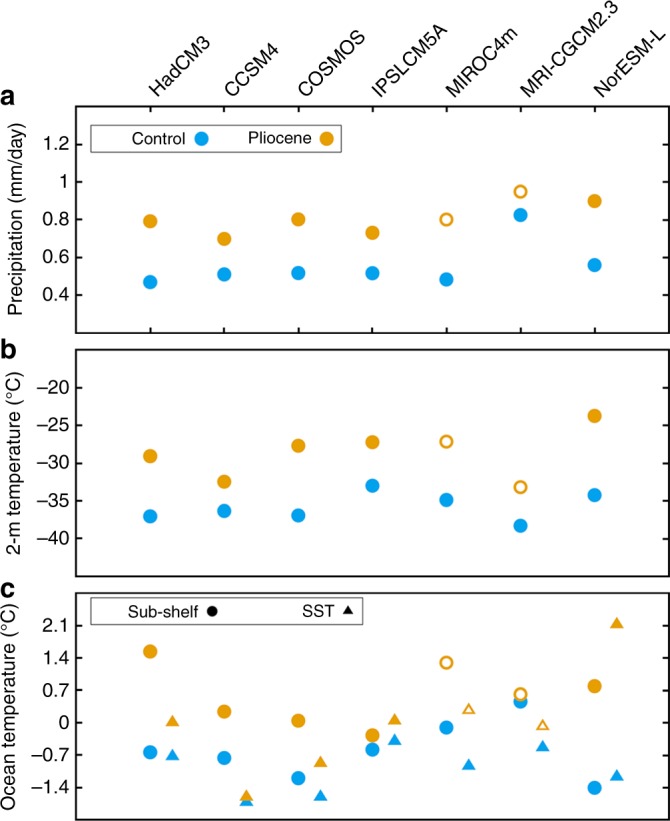
PlioMIP climatological averages. **a** Precipitation (mm/day), **b** near surface (2 m) temperature (°C) and **c** ocean temperature (for both the sea-surface and sub-shelf in °C) predicted by the PlioMIP climate models. Note that the sub-shelf temperatures have been extrapolated from the nearest ocean grid point in the GCM (due to the nature of the land-sea mask in the GCMs—see Methods and Supplementary Figs. 2 and 3). Unfilled circles and triangles (for MIROC4m and MRI-CGCM2.3) denote climatologies for the Pliocene that were not used to force ISMs presented in the main analysis in this study

Here, we use seven of the coupled atmosphere-ocean global climate models (GCMs) from PlioMIP Phase 1 (HadCM3^32^ as used in ref. ^28^, COSMOS^33^, CCSM4^34^, IPSLCM5A^35^, MIROC4m^36^, MRI-CGCM2.3^37^ and NorESM-L^38^; Supplementary Table 1) in order to provide climatological forcing for the ISMs. A summary of key climate model components can be found in ref. ^1^ and the differences in model resolution are shown in Supplementary Table 1. All of the PlioMIP1 models implemented PRISM3 boundary conditions for their mid-Pliocene experiment, which included a reduced AIS^26^ (see Methods). Near surface temperature differences over the AIS (including over ice shelves) simulated by the PlioMIP1 models range from +3.9 to +10.5 °C (relative to the pre-Industrial; Fig. 1; Supplementary Fig. 1). Circum-Antarctic ocean surface temperatures during the Pliocene are variable (between −1.6 and 2.1 °C higher than pre-industrial) with extrapolated sub-shelf temperatures reaching as much as 2.2 °C higher than pre-industrial (see Methods). Precipitation over Antarctica in general increases to around 0.8 mm day^−1^ in the Pliocene simulations from an average of 0.55 mm day^−1^ (Fig. 1; Supplementary Fig. 1). On average, Pliocene surface air temperatures over West Antarctica (WAIS) are predicted to be 15 °C warmer than East Antarctica (EAIS). The warmest areas of East Antarctica are around the Wilkes Land margin, in the region that was prescribed as ice-free in the PRISM3 boundary conditions (Supplementary Fig. 2).

### Control simulations of the modern Antarctic ice-sheet

**Fig. 2:**
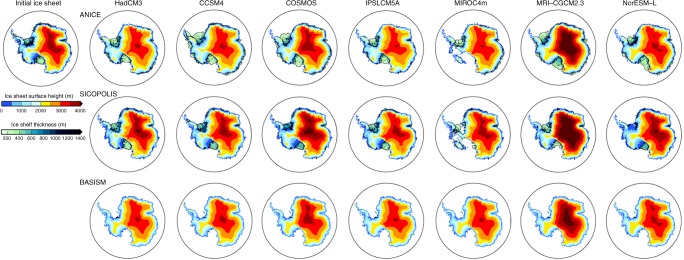
Simulated modern ice-sheets. Predicted modern ice-sheet surface height (m) and ice-shelf thickness (m) when using the climatological forcing fields from the PlioMIP climate models. Initial modern (Bedmap2) ice-sheet is shown in the top left for ref. ^39^. Note that ice-shelves are only simulated by the ANICE and SICOPOLIS ISMs

The climatological fields from the PlioMIP pre-industrial experiments are used to predict a modern AIS, which can be compared with the Bedmap2 reconstruction^39^. We use forcing fields from each of the seven PlioMIP climate models to run simulations with three ISMs, producing an ensemble of 21 control simulations. Whilst there is some consistency across the ISMs in the prediction of the grounded extent of the modern ice-sheet, especially over the EAIS (Fig. 2), there are considerable differences in the predictions of the extent of the WAIS and also the ice-shelves.

**Fig. 3:**
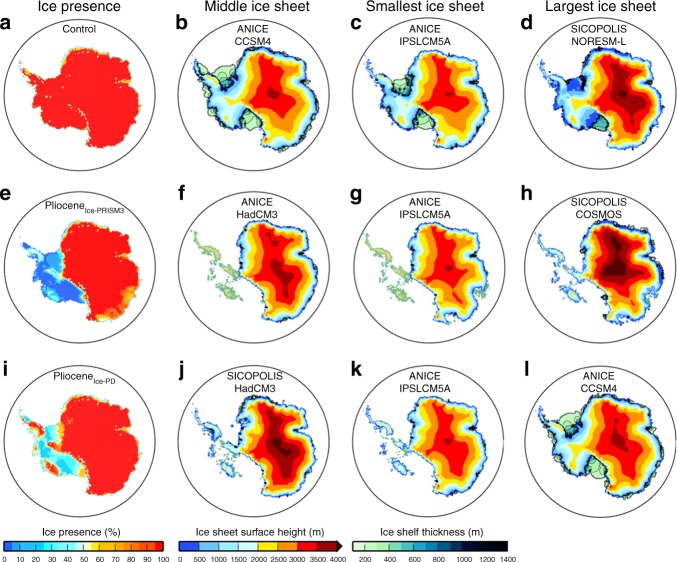
Summary of ice-sheet predictions. **a**, **e**, **i** Ice-sheet presence prediction for each of the climate scenarios (as a percentage of the total ensemble members). Also shown is the middle (**b**, **f**, **j**), smallest (**c**, **g**, **k**) and largest (**d**, **h**, **l**) ice-sheet configuration (surface height (m) and ice-shelf thickness (m)). Middle is the 5th ranking ice volume from the list of 10 SIA-SSA model results. Results from the control simulations of MIROC4m and MRI-CGCM2.3 are not included in this figure to allow better comparison between the presented scenarios

A visual comparison of the simulated modern ice-sheets highlights some GCM–ISM combinations that result in a poor reconstruction of the present day ice-sheet. The final simulated modern AIS grounded ice volume varies considerably between the models (23.64 × 10^6^–37.36 × 10^6^ km^3^), and is generally larger than the Bedmap2 reconstruction (26.54 × 10^6^ km^3^; volume difference between Bedmap2 and ISM predictions equates to a difference in sea level of between +6.3 m s.e. and −25.3 m s.e. (metres of sea level equivalent); ref.^39^; Fig. 3). In order that we only consider GCM–ISM combinations that provide a reasonable representation of the modern AIS in our Pliocene analysis, we developed performance criteria to assess predictions of the modern AIS, whilst also comparing our results to the Bedmap2 AIS reconstruction (see Methods and Supplementary Note 1). This leads to the removal of MRI-CGCM2.3 and MIROC4m from the Pliocene analysis. Whilst it is beyond the scope of the current study to fully investigate the potential reasons for a volumetrically divergent prediction of the present-day AIS, we examine the likely climate forcings driving this (see Supplementary Note 2).

It should be noted that the control simulations presented here were not tuned to give an optimal representation of the AIS using the different climate forcing fields and each ISM used only one set-up for each experiment (see Methods). This was done in order to allow for the assessment of model dependency of the results (without having to take into consideration the impact of potentially different ISM parameter value choices). We have shown that five out of the seven GCMs lead to a reasonable representation of present-day Antarctica. This gives us confidence when using the same model set-up to assess the climate model dependency of mid-Pliocene Antarctic simulations. For clarity, we have excluded two of the GCM–ISM combinations from our Pliocene analysis, however, the results from these GCM–ISMs are shown in Supplementary Figs. 11 to 13.

### Simulated mid-Pliocene ice-sheets

**Fig. 4:**
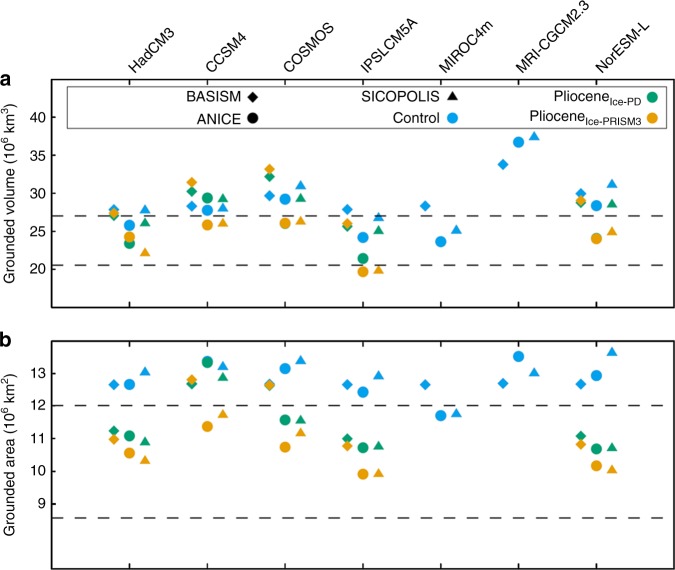
Simulated ice-sheet volume and area. Grounded ice-sheet volume (**a**; ×10^6^ km^3^) and area (**b**; ×10^6^ km^2^) prediction for each ISM given each of the climate model forcings for the Control, Pliocene_Ice-PD_ and Pliocene_Ice-PRISM3_ experiments. Horizontal dashed lines show the PRISM3 ice-sheet volume/area^26^ (bottom dashed lines) and the Bedmap2^39^ modern volume/area (top dashed lines). Note that the MIROC4m and MRI-CGCM2.3 climate models have not been used to force Pliocene scenarios presented here, however, the simulated ice-sheet volume and area for these GCM–ISM combinations can be seen in Supplementary Fig. 11

Mid-Pliocene ice-sheet simulations were initialised from the PRISM3 ice-sheet reconstruction (Pliocene_Ice-PRISM3_; Fig. 4), which is consistent with the boundary conditions applied within the PlioMIP GCMs^24^. Fifteen ensemble members were run, using temperature, precipitation and ocean temperatures from the five remaining PlioMIP climate models to force three ISMs. In general, there is a greater degree of variation between the individual ISM reconstructions for the mPWP in terms of ice presence than has been shown in the modern experiments (Figs. 3a, e and 4). In total, the reconstructed AIS grounded ice volume varies from 19.68 × 10^6^ km^3^ (ANICE using IPSLCM5A forcing) to 33.18 × 10^6^ km^3^ (BASISM using COSMOS forcing; Fig. 4). Over 85% of the simulations produced a reduction in grounded ice volume (13 of the 15 cases), compared to pre-industrial simulations, but only two cases resulted in an ice-sheet that was smaller than the initial PRISM3 reconstruction (Fig. 4).

**Fig. 5:**
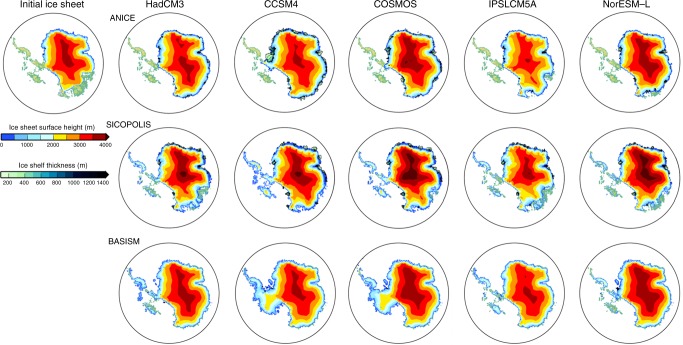
Simulated Pliocene ice-sheets initialised from PRISM3 Antarctica. ISM predictions of grounded ice-sheet surface height (m) and ice-shelf thickness (m) for the Pliocene_Ice-PRISM3_ experiments using the climatological forcing fields from the PlioMIP climate models. Initial PRISM3 ice-sheet is shown in the top left for ref. ^26^. Note that ice-shelves are only simulated by the ANICE and SICOPOLIS ISMs. Results from MIROC4m and MRI-CGCM2.3 are shown in Supplementary Fig. 12

When considering West and East Antarctica separately, we simulate a reduced WAIS relative to the present for all of the PlioMIP models using ANICE. SICOPOLIS predicts a consistent reduction in ice over WAIS, with every climate model forcing resulting in a collapse of the marine basins leaving just a number of isolated ice-caps on the West Antarctic islands (Fig. 5). The warmer-than-modern sub-shelf temperatures (Supplementary Fig. 3) prevent ice-shelves from growing around Antarctica in the majority of simulations using ANICE and SICOPOLIS (Fig. 5). BASISM shows the largest intra-model spread over West Antarctica, but as the only SIA model, it should be expected that the representation of ice over West Antarctica is not reliable.

Over East Antarctica, none of the climate models produce conditions that allow the ISMs to attain the extent of retreat prescribed in the PRISM3 ice-sheet reconstruction, due to a predominantly positive SMB over the Wilkes and the Aurora SGB driven largely by increased precipitation in these regions (Fig. 5 and Supplementary Figs. 2 and 4). Figure 3e shows strong agreement between models in the centre of East Antarctica, however, only around 40% of the ensemble members suggest ice-sheet reduction over the Wilkes and Aurora SGBs. With ANICE, the Wilkes and the Aurora SGBs only remain open-ocean in the simulations using IPSLCM5A and NorESM-L, and there are also small portions of coastline that continue to be ice-free (Fig. 5). With SICOPOLIS, there appears to be a much more consistent response in the Wilkes Land region whereby ice extends out from its initial PRISM3 grounding line, but does not entirely reach the modern coastline in any of the simulations (Fig. 5). BASISM predicts a modern ice extent for all climate scenarios over East Antarctica, suggesting that the inclusion of higher order and grounding line physics are key to simulating ice-sheet reduction in this region. By using different ISMs, we can see that in terms areal extent and the details of predicting ice conditions in certain SGBs, the result is highly dependent on the choice of ISM and not necessarily simply reliant on the choice of climate model forcing.

A final observation for the Pliocene_Ice-PRISM3_ ensemble is the large range of ice-sheet thickness predictions over the EAIS for the mPWP (Supplementary Fig. 5). Whilst this is anticipated in the regions where there is low agreement amongst the models regarding ice presence (e.g. Wilkes and Aurora SGBs; Fig. 3e), over the continental interior we still demonstrate thickness differences of over 1.5 km. The range in GCM-predicted temperatures and precipitation rates over the EAIS corresponds to a large range in SMB in this region (varying from around 600 to 1800 Gt year^−1^), which is a likely contributor to broad differences in ice-sheet thickness in the centre of East Antarctica.

### Sensitivity to GCM boundary and ISM initial conditions

**Fig. 6:**
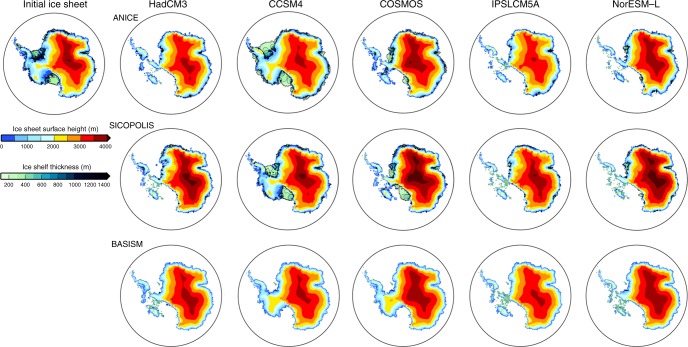
Simulated Pliocene ice-sheets initialised from present-day Antarctica. ISM predictions of grounded ice-sheet surface height (m) and ice-shelf thickness (m) for the Pliocene_Ice-PD_ experiments using the climatological forcing fields from the PlioMIP climate models. Initial present-day ice-sheet is shown in the top left for ref. ^39^. Note that ice-shelves are only simulated by the ANICE and SICOPOLIS ISMs. Results from MIROC4m and MRI-CGCM2.3 are shown in Supplementary Fig. 12

One feature of the PlioMIP1 experimental design, based on the PRISM3 reconstruction of boundary conditions, is the specification of reduced ice-sheet volume and extent for the mPWP. The PRISM3 ice-sheet reconstruction was in line with sea level records that were published at the time the project commenced and was also based on some of the initial ISM reconstructions of the mPWP^19,26^. It can be argued that such a prescribed retreat in the climate modelling simulations will lead to the ISMs predicting a greater level of ice reduction than may have occurred in the warmest intervals of the Pliocene. However, to have prescribed a modern AIS in the PlioMIP1 GCMs would have also been incorrect, as this would have been incompatible with various lines of proxy evidence (e.g. sea level data). Ultimately, in the absence of reliable geological reconstructions of mPWP ice-sheets a choice must be made of how to initialise climate model simulations. It is nevertheless useful to test the sensitivity of our mid-Pliocene simulations to the prescribed PRISM3 ice-sheet in the climate models. It is not possible for each GCM to repeat their PlioMIP1 experiment with a different AIS boundary condition (e.g. modern), therefore we have analysed whether or not the initial conditions prescribed in the ISM impact upon the predicted result. We ran a suite of simulations where each ISM experiment was initialised from the Bedmap2 modern ice-sheet configuration (Pliocene_Ice-PD_; Figs. 4 and 6). This provides a good test of whether or not the models can simulate a retreat from the present-day grounding line, given the imposed Pliocene climate forcing.

In terms of ice-sheet extent, most of the three ISMs showed a similar pattern of large-scale response, in that the Pliocene_Ice-PD_ equilibrium AIS was larger than the Pliocene_Ice-PRISM3_ ice-sheets, but smaller than the modern reconstruction (Figs. 4 and 6). In the SIA-SSA ISMs ocean conditions are warm enough to melt the majority of the ice shelves and reduce ice to the islands of West Antarctica for all of the simulations except those using forcing fields from CCSM4 (Fig. 6). ANICE displays a strong hysteresis over West Antarctica, as under the same climate conditions ice remains on the West Antarctic islands in Pliocene_Ice-PD_, whereas it is absent in the Pliocene_Ice-PRISM3_ experiments. For the EAIS most models produce a similar surface topography to Pliocene_Ice-PRISM3_ when using modern starting conditions, with the exception that no significant retreat is demonstrated in any model in the Wilkes and Aurora SGBs (Fig. 6). This suggests that a modern sized EAIS would require additional forcing to retreat from a modern size than the standard PlioMIP experimental design, e.g. stronger insolation forcing and/or even higher concentrations of atmospheric CO_2_, when using SSA–SIA ISMs. Alternatively, this could highlight the potential need to include alternative grounding line physics within the ice-sheet modelling framework (e.g. ref. ^21^).

In terms of volume, BASISM and ANICE tend to predict ice-sheets for Pliocene_Ice-PD_ that are relatively close to their predicted Pliocene_Ice-PRISM3_ volume (Fig. 4). Predictions from the SICOPOLIS model are often more divergent with the Pliocene_Ice-PD_ being larger than the Pliocene_Ice-PRISM3_ by an average of 3.8 × 10^6^ km^3^. This is most likely attributable to the different ways in which the ISMs compute SMB. In particular, the method of converting total precipitation to effective accumulation is different between the ISMs.

### Predicted sea level changes

**Fig. 7:**
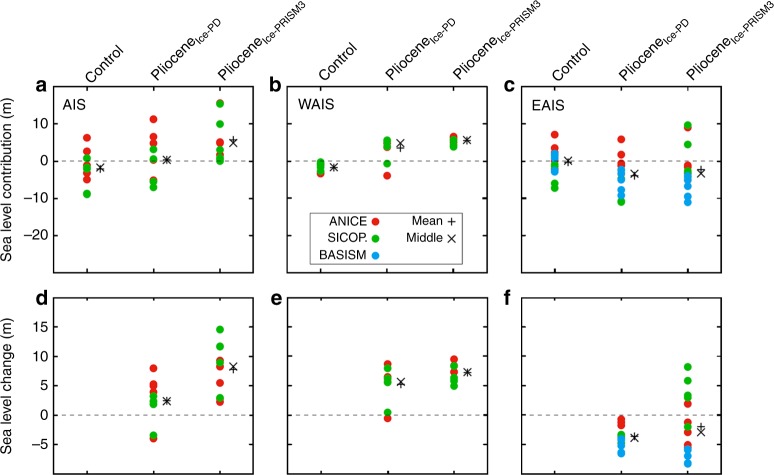
Sea level contributions. Sea level contribution relative to Bedmap2^39^ of the total ice volume of the **a** AIS, **b** WAIS and **c** EAIS. The relative change in sea level for the two Pliocene experiments (Pliocene – Control experiment shown in panels **a**–**c**) for **d** AIS, **e** WAIS and **f** EAIS. Since BASISM is a SIA-only model, results are only shown for the EAIS. Middle is the 5th ranking sea level contribution from the list of 10 SIA-SSA model results. There are no results shown in this figure for the MIROC4m or MRI-CGCM2.3 predictions, however these can be seen in Supplementary Fig. 13

The contributions of Antarctica to sea level are shown in Fig. 7 (see Methods for sea level calculations). On average the 10 SIA-SSA simulations, which have also been used in the Pliocene analysis overestimate the present-day ice volume by 2.13 m s.e. for the control simulations relative to Bedmap2 (Fig. 7a). For the mPWP, we consider sea level contribution for the whole of Antarctica for Pliocene_Ice-PRISM3_ and Pliocene_Ice-PD_ relative to Bedmap2 and as a change from the control prediction using the same climate model forcing for each ISM (Fig. 7a, d; Table 1). We focus on our predictions relative to the control prediction from each ensemble member as this goes some way to reduce any biases inherent in our modern simulations, i.e. the too large modern ice-sheet predictions. Our results show that mean sea level change relative to the control simulation (calculated from SIA-SSA simulations only) is 7.79 ± 4.06 m s.e. (1 standard deviation, Pliocene_Ice-PRISM3_) and 2.43 ± 3.53 m s.e. (1 standard deviation, Pliocene_Ice-PD_; Fig. 7d). For Pliocene_Ice-PD_ an average sea level rise of 5.24 m s.e. from the WAIS is partially offset by a sea level fall from the EAIS (−2.80 m s.e.; Fig. 7e) when considering only SIA-SSA ISMs. Similarly, the EAIS shows lower sea levels than the control (mean of −1.99 m s.e.) when considering the entire Pliocene_Ice-PRISM3_ ensemble.

**Table 1:** Uncertainties in the prediction of sea level

Source of uncertainty considered	Maximum ΔSL	Minimum ΔSL	Range in ΔSL	Mean ± SD	Reference
ISM dependency (PRISM3 initial conditions)	13.79	7.66	6.13	9.76 ± 2.13	de Boer et al.^28^
ISM dependency (PD initial conditions)	2.90	−11.80	14.70	−1.89 ± 4.93	
ISM parameterisations relating to surface melting, ice flow and sub-shelf melting	6.6	−4.2	10.8	–	Yan et al.^43^
Pliocene temperature and precipitation	13.0	6.2	6.8	–	
Subgrid scale melt parameterisations at grounding line (alongside temperature biases of 1 and 2 degrees in the regional climate model)—All simulations	–	–	–	8.57 ± 2.8	Golledge et al.^16^
As above but with no subgrid scale melting	–	–	–	7.44 ± 1.95	
As above but including subgrid scale melting	–	–	–	9.7 ± 3.15	
Parameterised mechanisms for ice cliff failure and ice-shelf hydrofracturing under a warm Pliocene-like scenario	~18	~1.6	–	–	Pollard et al.^22^
Exploration of parameter values within one ISM that control the relationship between ocean temperature and sub-ice-shelf melt rates, hydrofracturing and maximum rates of marine-terminating ice-cliff failure	12.41	3.52	8.89	–	DeConto and Pollard^21^
Dynamic topography	7.4	5.4	1.7	–	Austermann et al.^44^
Bedrock topography	17.9	12.6	5.3	–	Gasson et al.^45^
GCM dependency (Pliocene_Ice-PRISM3_)	14.57	2.24	12.33	7.79 ± 4.06	This study
GCM dependency (Pliocene_Ice-PD_)	7.97	−3.99	11.96	2.43 ± 3.53	
GCM dependency (all experiments)	14.57	−3.99	18.56	5.11 ± 4.65	
Impact of orbital forcing on climate and ice sheet predictions within a SIA ISM framework	13.52 (EAIS only)	−7.14 (EAIS only)	20.66 (EAIS only)	–	Dolan et al.^20^

Summary of previous ice-sheet modelling predictions of Pliocene global mean sea level change relative to present-day due to Antarctic ice-sheet changes in terms of range (maximum and minimum contributions) and as a mean value. The type of uncertainty addressed in each study is also summarised

## Discussion

In order to be confident in model predictions, it is critical to quantify the impact of various sources of uncertainty on palaeo ice-sheet reconstructions. The quantification of uncertainty introduced by the choice of climate model or ISM used has been the primary goal of the PLISMIP project presented here and in previous papers^28,40,41^.

Our results indicate a high level of climate model dependency in reconstructing the Antarctic ice-sheet volume (Fig. 4) and resulting sea level change (Fig. 7). Given the complex nature of the PlioMIP1 climate models used to provide our forcing fields, understanding the reasons for differences in the ISM predictions is non-trivial. It is clear that some of the major variations in the simulated extent and volume of the AIS for the Pliocene scenarios are closely linked to the way temperature, precipitation and ocean temperatures are predicted among the climate models. Warming among the PlioMIP1 climate models in the Antarctic region has been previously attributed to the changes in the cryosphere and surface albedo^42^. Over Antarctica, the clear sky albedo is the dominant component of the energy balance, which leads to increased warming in all PlioMIP models south of 75°S (although there is a large range in warming at 90°S (1.5–12 °C))^42^.

The difference in the results from the Pliocene_Ice-PRISM3_ ensemble (Fig. 5) and the Pliocene_Ice-PD_ ensemble (Fig. 6) also shows the level to which the equilibrium ice-sheet predictions are dependent upon the history of the ice-sheet system (hysteresis); in this case, the specific initial conditions used in the ISM set-up. This is most clearly demonstrated in the results using ANICE and SICOPOLIS driven by the CCSM4 climate model. CCSM4 exhibits some of the coldest sub-shelf ocean temperatures, especially over the Ross Ice Shelf (Supplementary Fig. 3), and has the coldest temperatures (~−25 °C) over West Antarctica of the considered PlioMIP models (Supplementary Fig. 1). This may be in part due to the higher albedo levels in CCSM4 (*α* = ~0.6) over West Antarctica relative to the other GCMs helping to promote cooling. In the Pliocene_Ice-PRISM3_ simulation, the precipitation rates over West Antarctica are not sufficient to create a strong positive SMB (Supplementary Fig. 4), thus the results from CCSM4 are in line with the other GCMs (in that the Pliocene WAIS remains collapsed from the initial PRISM3 starting configuration; Fig. 5). However, when the ISMs are initialised with a modern AIS (Pliocene_Ice-PD_; Fig. 6), hysteresis is strong due to the fact that the combination of lapse-rate corrected temperatures that are applied and ocean-driven melting are not sufficient to cause a reduction of the WAIS and associated retreat of the ice shelves in either ISM.

As we have shown a broad spread in sea level changes associated with our ensemble of AIS predictions (Fig. 7d), it is useful to understand how these compare to sea level data and also other modelling studies for the mPWP in order to assess which of a number of sources of uncertainty lead to the largest range in predictions of the AIS (Table 1). The results presented here indicate that the spread in sea level change predictions for the mPWP from the PlioMIP ensemble (based on the difference to the modern volume) using the SIA-SSA models is between 2.24 m s.e. (ANICE with HadCM3) and 14.57 m s.e. (SICOPOLIS with IPSLCM5A) for the Pliocene_Ice-PRISM3_ scenario (the mean is 7.79 m s.e.). Using just the ANICE ISM, the range in sea level change predictions is 7.04 m s.e. for the Pliocene_Ice-PRISM3_ simulations, and for the SICOPOLIS ISM the range is slightly larger (11.64 m s.e.; Fig. 7d). However, the average difference between the predictions of the two SSA–SIA ISMs using one climate model forcing is only 4.35 m s.e., which shows that the results are more dependent upon the chosen climate model forcing and not the ISM used. However, both have an impact on the determined result. The range in mean sea level change predicted here in both of our Pliocene scenarios is 14.57 to −3.99 m s.e. using only SIA-SSA ISMs. This demonstrates that the choice of initial conditions imposed in the ISMs is an important control on the total range of sea level predictions, as demonstrated previously^28^.

Focussing primarily on studies employing SIA-SSA models, Table 1 provides a summary of previous work assessing various sources of uncertainty in predicting the Pliocene AIS (see also ref. ^16^). Following a similar experimental design to that presented here and initialising from a PRISM3 ice-sheet, ref. ^28^ predicted a range in mean sea level rise for equilibrium Pliocene ice-sheets of 5.83 m s.e. (7.96–13.79 m s.e.) when considering six different SIA-SSA ISMs but only one climate model forcing (HadCM3). The comparable range shown here incorporating five different GCMs is 12.33 m s.e., supporting the conclusion that climate model dependency on AIS predictions for the Pliocene is high.

Yan et al. perturbed ISM parameterisations (positive degree-day (PDD) factors for ice and snow, enhancement flow factors for the SIA and SSA velocities and the sub-shelf melt parameter) using the Parallel ISM (PISM) driven by climatological forcing from the NorESM climate model^43^. They simulated AIS configurations ranging from −4.2 to 6.6 m s.e. volume (causing a 10.8 m s.e. variation due to parameter uncertainty). In the same study, temperature and precipitation were also increased in steps of 1 °C for temperature and 5.1% increase in precipitation per degree of temperature change in order to explore the influence of uncertainty in the Pliocene climate forcing on the modelled AIS. Considering this additional warming, the mass loss of the AIS relates to a global mean sea level rise of 6.2–13.0 m relative to present day (range of 6.8 m s.e.; ref. ^43^). The impact of uncertainties in dynamic topography^44^ and Antarctic bedrock topography^45^ have also been considered in a Pliocene ISM framework, however, these mechanisms only cause a small variation in predicted AIS volume and hence mean sea level change (Table 1). Therefore, it is clear from our results that in terms of the range of predictions of global mean sea level change, the climate model chosen has the potential to exert a larger influence on the results than the ISM, ISM parameterisations or changes in topography.

While ideally proxy-based estimates of global mean sea level change for the mPWP could be used to constrain which of the modelled AIS scenarios is more or less likely, significant uncertainty remains in the estimates of Pliocene sea level high stands^4,46^. Although in general all data suggest a higher-than-present sea level indicative of a reduced Greenland ice-sheet and retreated AIS^10,12^, estimates range from between 10 and 30 m above present-day^4–8^, based on a range of global proxy records (e.g. palaeoshorelines), for which the largest uncertainties arise from considering processes such as mantle convection and glacial isostatic adjustment^4,5,9,47^. Recent studies inferring sea level changes from oxygen isotopes have offered a more constrained reconstruction of Pliocene sea level, suggesting that high-stands were between 9 and 13.5 m above modern, implying that the EAIS may be less sensitive during the Pliocene than previously considered^48^. A further study using a novel approach to constrain mPWP sea level high stands by assessing whether or not particular AIS configurations are consistent with the oxygen isotope record have suggested that AIS contribution to mPWP sea level rise was at most ~13 m^46^. However, a recent review^49^ of the sources of uncertainty associated with using marine geochemical proxies to infer past changes in sea level (e.g. refs. ^46,48^), has shown that diagenesis and potential long-term changes in seawater chemistry likely have large and poorly constrained effects on interpretations of such records. This may limit their ability to make meaningful Pliocene sea level estimates at the present time^49^.

The question as to whether or not the EAIS was dynamic or reduced in the Pliocene is a long-standing issue (see ref. ^19^). Our results show that in around 40% of the simulations for the Pliocene_Ice-PRISM3_ experiment, substantial ice-sheet reduction is exhibited in the Wilkes and Aurora SGB and around Wilkes Land (Figs. 3e and 4). This is consistent with ice-rafted debris data thought to be indicative of a dynamic Wilkes Land margin region during the Late Pliocene^12,13^. However, this result depends on the boundary conditions imposed in the climate model^28^ and the starting conditions of the ISMs. For example, none of the AIS configurations presented in the Pliocene_Ice-PD_ experiments suggest significant retreat into the EAIS SGBs (Fig. 6). It must be acknowledged that this is potentially inconsistent with geological proxy evidence for retreat of portions of the EAIS during the mPWP (e.g. ref. ^12^). Nevertheless, the results presented here also demonstrate that, while the total AIS volume is most heavily influenced by the choice of climate model, detailed predictions of areal extent are more likely to be influenced by the ISM used and ISM initial conditions (Figs. 5 and 6). For example, Fig. 5 shows that ANICE predictions generally would lead to the conclusion that the Aurora SGB was covered in ice (apart from when using IPSL-CM5A climate forcing), BASISM predictions always suggest an ice-covered region, whereas SICOPOLIS predicts some ice retreat under all climatologies.

There are also additional sources of uncertainty that have been shown previously to impact upon the details of predicted SGB retreat that have not been considered here. When considering the modern AIS under future warming scenarios, it has been shown that the implementation of subgrid interpolation of basal melting at grounding lines within SIA-SSA ISMs may accelerate grounding line retreat^50^ and impacts upon the final ice volume predicted in long palaeo-integrations increasing the range in volume predictions^16^. None of the ISMs considered here include fine scale representations of the grounding line, for example, subgrid friction schemes^51^ or adaptive mesh refinement^52^ that has been shown to improve accuracy in a modern setting at very high (<1 km) resolution. DeConto and Pollard also employ a parameterisation of ice-shelf hydrofracturing and ice-cliff collapse of marine-terminating ice margins, which is not included in any of the ISMs here and this could potentially have a large impact on predictions of sea level change for the Pliocene^21,22^ (Table 1). As ISMs that are applicable to palaeo problems evolve (e.g. can be run on a continental scale for long time integrations), it is likely that such fine scale and novel grounding line parameterisations will continue to alter our understanding of potential ice-sheet collapse in the EAIS SGBs and this will be an interesting avenue for future research.

PLISMIP was the first ISM intercomparison project set up to shed light of palaeo ice-sheet variability, with the ambition of helping to understand the sensitivity of the ice-sheets in a warmer-than-modern world^27^. To realise this, one of the key aims of the PLISMIP project was to reconstruct the most likely geometry and volume of ice masses on Greenland and Antarctica. The results over Greenland allowed for an improvement to be made in the understanding of the potential extent and configuration of the Greenland Ice Sheet during the Pliocene^40,41^ and a new reconstruction has now been incorporated into the PRISM4 Pliocene reconstruction^53^ and the experimental design for PlioMIP2^54^. However, results presented here and in other ice-sheet modelling studies for the Pliocene AIS^28^ show that the level of uncertainty associated with simulating a marine-based WAIS and also the potential dynamic nature of the grounding line over the EAIS mean that we cannot be confident in using the results from this study to infer a most likely AIS configuration for the mPWP.

Nevertheless, our results unequivocally demonstrate that the climate forcing fields used (and in effect the climate model chosen) can have a significant impact on the predicted equilibrium state ice-sheet for the mPWP. When compared against other sources of uncertainty (e.g. ISM parameterisations and the choice of ISM), the atmospheric forcing field used brings in the largest source of uncertainty in terms of the global mean sea level change associated with volumetric changes in the ice-sheet (Table 1). We also show, however, that the details of the predicted ice-sheet configuration are generally more affected by which ISM is used, rather than the particular climate forcing, with a caveat that this may be affected by some of the ice boundary condition choices used within the PlioMIP models. Therefore it is important that as a community we do not place undue confidence on the ice-sheet reconstructions of one ISM or one climate model, when additional sources of structural uncertainty have not been considered.

## Methods

### Ice-sheet model forcing

Surface temperature, precipitation, ocean temperature and topography (Supplementary Fig. 1) were taken from results of the coupled-atmosphere ocean climate simulations run as part of the PlioMIP project. Full details for the experimental design of PlioMIP can be found in ref. ^27^. Briefly it involved the implementation of PRISM3 boundary conditions^26^ in IPCC AR4 class general circulation models (GCMs). PRISM3 boundary conditions included increased atmospheric CO_2_ concentrations, an altered palaeogeography, including changes to the land-sea mask (LSM), a changed vegetation cover^55^ and altered Pliocene ice-sheet configurations. In PRISM3 West Antarctica is deglaciated and East Antarctica is retreated beyond the marine portions of the Wilkes and Aurora SGB^56^. Where possible, modelling groups were asked to alter their LSM to allow for a West Antarctic Seaway. More details of the seven GCMs that have been used in this study and their implementation of the PRISM3 boundary conditions can be found in Supplementary Table 1 and the references therein.

### Experimental design PLISMIP

The experimental design of the simulations follows the same criteria as outlined in ref. ^27^ and presented in ref. ^28^. Here, we ran simulations with three different ice-sheet model focussing on the equilibrium response of the ice-sheet to seven different climate models from the PlioMIP ensemble^1^. An ensemble of simulations was performed for three different scenarios. The first scenario was a control experiment with climate forcings from the GCMs that were run with pre-industrial boundary conditions. The second was a Pliocene_Ice-PRISM3_ experiment with climate forcings from the GCMs that were run with Pliocene boundary conditions. The ice-sheet models were initialised with the PRISM3 ice-sheet topography^28^. The final scenario was a Pliocene_Ice-PD_ experiment with climate forcings from the GCMs that were run with Pliocene boundary conditions. The ice-sheet models were initialised with the PD ice-sheet topography from Bedmap2^39^.

The seven Atmosphere-Ocean GCMs that we use here are outlined in Supplementary Table 1. For all simulations, an initial spin-up procedure for ice-sheet temperatures is employed for which ice-sheet topography and surface temperature of the ice-sheet stay constant over 100 000 years. For the subsequent 100 000 year equilibrium run, the surface temperature from the GCM is corrected with a lapse rate of −8 °C km^−1^ for the difference between the modelled surface elevation and the reference topography in the GCM:

1 $$T_{{\mathrm{surf}}}\left( t \right) = T_{{\mathrm{GCM}}} - 0.008 \times \left( {H_{{\mathrm{surf}}}\left( t \right) - H_{{\mathrm{GCM}}}} \right).$$

Here, *T*_surf_ is the temperature at the surface of the ice-sheet and *T*_GCM_ is the temperature of the GCM in °C. The surface elevation in metres of the ice-sheet and of the GCM are given by *H*_surf_ and *H*_GCM_, respectively. For all experiments, we use monthly averaged climatologies of the GCMs. It should also be noted that the ISMs reached equilibrium after the first 30 000 years of the simulation.

Simulations with ANICE and SICOPOLIS are performed including land-based and floating ice, whereas for the BASISM simulations only land ice is included in the simulations. For land ice all three models include the shallow ice approximation (SIA), which approximates the stress balance by only taking into account horizontal shear stress and lead to velocities that are dependent on depth^57^. On the contrary, for floating ice we apply the SSA that only includes longitudinal stress and vertically independent horizontal velocities^58^.

### Interpolation of climate fields

All ice-sheet models are run on a 40 km by 40 km grid of 141 × 141 grid points. All climate fields from the GCMs, i.e. surface-air temperature, precipitation, ocean temperature and reference topography, are interpolated on the ice-sheet rectangular grid using OBLIMAP v2.0. A polar stereographic projection is employed, using a central longitude of 0°E and a standard parallel of 24.7°, i.e. a latitude of true scale of 65.3°S^59^. For the 3-D ocean temperatures, we use a distance weighting scheme to extrapolate temperatures to underneath the ice shelves, where ocean temperatures are largely unresolved in the GCMs.

### Assessment of GCM–ISM performance for the modern AIS

All of the control ISM simulations were assessed in terms of how well they reconstruct the modern AIS (when compared to Bedmap2^39^ (see Supplementary Note 1 and Supplementary Figs. 7–9)). Additionally, we have considered each GCM individually to assess whether the predicted volumes (from the three ISMs) fall within the range of the mean plus or minus the standard deviation of the other models. If we assume a normal distribution, we would expect, on average 68% of predicted volumes to fall within this range. In cases where only one or no present-day simulations are within one standard deviation of the mean, it suggests that this particular GCM is producing ice-sheets significantly different from the others and is skewing the ensemble means (Supplementary Table 2 and S3). Where a GCM falls outside of the ensemble mean (±1 standard deviation) and also demonstrates poor skill in reconstructing elements of either volume, area or ice-sheet thickness the GCM has been excluded (see Supplementary Note 1). Nevertheless, for completeness and to demonstrate the effect of including all GCM–ISM combinations, all results are shown in Supplementary Figs. 11 to 13.

### Calculating contributions to sea level

The sea level contribution from each modelled ice-sheet is calculated after a run is finished, summing the ice thickness above flotation and including a correction for the change in bedrock topography:

2 $$\Delta S =	 \left( {\mathop {\sum}\nolimits_{i,j} {Hi_{0{\mathrm{af}}} - Hi_{{\mathrm{af}}} + {\mathrm{min}}\left( {0,Hb} \right) - {\mathrm{min}}\left( {0,Hb_0} \right)} } \right) \\ 	 \times 40000 \times 40000/O_{{\mathrm{area}}}.$$

Here, *Hi*_0af_ and *Hi*_af_ are the ice thickness above flotation for the initial Bedmap2 ice-sheet and the final modelled ice-sheet in metres water equivalent, respectively:

3 $$Hi_{{\mathrm{af}}} = \frac{{\rho _{\mathrm{i}}}}{{\rho _{\mathrm{w}}}}Hi + Hb.$$

*Hi* is the ice thickness in metres and *Hb* is the bedrock topography in metres below sea level. Parameters are given in Supplementary Table 4. Ice-sheet volumes and sea level contributions shown here include an area correction following the methods described in ref. ^60^.

### The ANICE ice-sheet model

The ANICE ice-sheet model is a 3-D thermo-mechanical finite difference ice-sheet model and is part of the IMAU-ICE (Institute for Marine and Atmospheric Research Utrecht) ice-sheet model package. The model we use here is the same version as has been used in previous publications^28,61^. In ANICE, ice velocities are calculated on land with the SIA and uses the SSA for sliding, i.e. ice streams and shelf velocities. A Mohr–Coulomb plastic law paramaterisation is employed to derive basal stresses that are used to derive the SSA velocities on land^61,62^.

The surface mass balance is determined with monthly fields of precipitation, temperature and insolation at the top of the atmosphere. The precipitation is adjusted with surface temperature. Surface melting is calculated with an insolation temperature melt model, for which a linear relation is used for temperature combined with the net incoming short-wave radiation, using an internally calculated albedo^61^. Sub-shelf melting underneath the ice shelves is included as a heat-transfer equation^63^:

4 $$M_{{\mathrm{shelf}}} = \rho _{\mathrm{w}}c_{{\mathrm{pO}}}\gamma _{\mathrm{T}}F_{{\mathrm{melt}}}\left( {T_{{\mathrm{oc}}} - T_{\mathrm{f}}} \right)^n/L\rho _{\mathrm{i}}.$$

The different parameters are described in Supplementary Table 4. For ANICE, *n* = 1. Here, *T*_oc_ is the ocean temperatures of each GCM that are vertically interpolated to the depth of the ice shelves and *T*_f_ is the freezing temperature^63^:

5 $$T_{\mathrm{f}} = 0.0939 - 0.057\cdot S_{\mathrm{O}} + 7.64 \times 10^{ - 4}z_{\mathrm{b}},$$

with *S*_O_ is the mean value for the salinity of the ocean and *z*_b_ is the bottom of the ice-shelf below sea level. The different parameters are described in Supplementary Table 4. For exposed ice-shelves and open ocean grid points, additional melt rates of 3 and 5 m year^−1^ are included, respectively. No additional calving law is applied.

### The SICOPOLIS ice-sheet model

SICOPOLIS (SImulation COde for POLythermal Ice-sheets) is a 3-D thermo-mechanical finite difference ice-sheet-shelf model^29^. On land, the model utilises a hybrid combination of the SIA and the SSA (including basal drag) as described in ref. ^30^, following ref. ^62^.

Basal sliding is described by a Weertman-type power law with a cubic dependence on basal shear stress, allowing sub-melt sliding following ref. ^64^. The distribution of basal sliding coefficients used in all runs is obtained from a calibration run using an iterative technique^17,30^.

For the surface mass balance, precipitation is converted to snow accumulation with a linear function of surface temperatures^65^. Surface melting is parameterised by a PDD method^66,67^. PDD factors are 8 and 3 mm day^−1^ °C^−1^ for ice and snow, respectively.

Ice-shelf basal melting is computed using Eq. (4)^68^, with *n* = 2. For the control simulations, a tuning melt factor of *F*_melt_ = 0.2 m s^−1^ K^−1^ is applied at the floating side of some grounding lines (northwest of Ronne Ice Shelf and south of Amery Ice shelf) to better represent their modern positions. At each time step, the calving front is iteratively checked for grid points with a thickness below 50 m, which are calved out, until no more calving occurs.

### SIA-only model BASISM

BASISM (British Antarctic Survey Ice-sheet Model) is a finite difference thermomechanical 3-D ice-sheet model, only including land-based ice using the SIA^31,56^. Basal sliding is not included in the model. Here, the model is free to grow to the extent of the modern grounding line.

An exponential function is used to convert temperatures into the number of PDDs^66^, which shows a high level of correlation between warmest month temperatures and observations of present-day melt^56^. This assumes that melt is determined by temperature variations alone and uses separate PDD factors for ice and snow melt and a maximum fractional refreezing rate. Here, standard parameters are set to 8 and 3 mm day^−1^ °C^−1^ for ice and snow melt, respectively^69^.

BASISM has been used in a number of previous mid-Pliocene modelling studies^19,20,40,56,70^ and therefore provides a frame of reference against which the more complex SSA–SIA hybrid models can be compared.

### Data availability

Data available on request from the authors.

## Electronic supplementary material

Supplementary Information

## References

[1] Haywood AM (2013). Large-scale features of Pliocene climate: results from the Pliocene Model Intercomparison Project. Clim. Past.

[2] Badger MPS, Schmidt DN, Mackensen A, Pancost RD (2013). High-resolution alkenone palaeobarometry indicates relatively stable pCO_2_ during the Pliocene (3.3–2.8 Ma). Philos. Trans. R. Soc. Lond. A.

[3] Martinez-Boti MA (2015). Plio-Pleistocene climate sensitivity evaluated using high-resolution CO_2_ records. Nature.

[4] Raymo ME, Mitrovica JX, O’Leary MJ, DeConto RM, Hearty PJ (2011). Departures from eustasy in Pliocene sea-level records. Nat. Geosci..

[5] Rovere A (2014). The Mid-Pliocene sea-level conundrum: glacial isostasy, eustasy and dynamic topography. Earth Planet. Sci. Lett..

[6] Rohling EJ (2014). Sea-level and deep-sea-temperature variability over the past 5.3 million years. Nature.

[7] Dutton, A. et al. Sea-level rise due to polar ice-sheet mass loss during past warm periods. *Science* **349**, aaa4019 (2015). 10.1126/science.aaa4019 26160951

[8] Miller KG (2012). High tide of the warm Pliocene: implications of global sea level for Antarctic deglaciation. Geology.

[9] Masson-Delmotte, V. et al. In *Climate Change 2013: The Physical Science Basis. Contribution of Working Group I to the Fifth Assessment Report of the Intergovernmental Panel on Climate Change* (eds Stocker, T. F. et al.) Ch. 5 (Cambridge University Press, 2013).

[10] Naish T (2009). Obliquity-paced Pliocene West Antarctic ice sheet oscillations. Nature.

[11] Cook CP (2014). Sea surface temperature control on the distribution of far-traveled Southern Ocean ice-rafted detritus during the Pliocene. Paleoceanography.

[12] Cook CP (2013). Dynamic behaviour of the East Antarctic ice sheet during Pliocene warmth. Nat. Geosci..

[13] Williams T (2010). Evidence for iceberg armadas from East Antarctica in the Southern Ocean during the late Miocene and early Pliocene. Earth Planet. Sci. Lett..

[14] Yamane M (2015). Exposure age and ice-sheet model constraints on Pliocene East Antarctic ice sheet dynamics. Nat. Commun..

[15] Pollard D, DeConto RM (2009). Modelling West Antarctic ice sheet growth and collapse through the past five million years. Nature.

[16] Golledge NR (2017). Antarctic climate and ice-sheet configuration during the early Pliocene interglacial at 4.23 Ma. Clim. Past.

[17] Pollard D, DeConto RM (2012). Description of a hybrid ice sheet-shelf model, and application to Antarctica. Geosci. Model Dev..

[18] de Boer B, Stocchi P, van de Wal RSW (2014). A fully coupled 3-D ice-sheet–sea-level model: algorithm and applications. Geosci. Model Dev..

[19] Hill, D. J., Haywood, A. M., Hindmarsh, R. C. M. & Valdes, P. J. In *Deep-Time Perspectives on Climate Change: Marrying the signal from Computer Models and Biological Proxies* (eds Williams, M. et al.) 517–538 (The Micropalaeontological Society Special Publications, The Geological Society, 2007).

[20] Dolan AM (2011). Sensitivity of Pliocene ice sheets to orbital forcing. Palaeogeogr. Palaeoclimatol. Palaeoecol..

[21] DeConto RM, Pollard D (2016). Contribution of Antarctica to past and future sea-level rise. Nature.

[22] Pollard D, DeConto RM, Alley RB (2015). Potential Antarctic Ice Sheet retreat driven by hydrofracturing and ice cliff failure. Earth Planet. Sci. Lett..

[23] Mengel M, Levermann A (2014). Ice plug prevents irreversible discharge from East Antarctica. Nat. Clim. Change.

[24] Haywood AM (2011). Pliocene Model Intercomparison Project (PlioMIP): experimental design and boundary conditions (Experiment 2). Geosci. Model Dev..

[25] Flato, G. et al. In *Climate Change 2013: The Physical Science Basis. Contribution of Working Group I to the Fifth Assessment Report of the Intergovernmental Panel on Climate Change* (eds Stocker, T. F. et al.) Ch. 9 (Cambridge University Press, 2013).

[26] Dowsett HJ (2010). The PRISM3D paleoenvironmental reconstruction. Stratigraphy.

[27] Dolan AM, Koenig SJ, Hill DJ, Haywood AM, DeConto RM (2012). Pliocene Ice Sheet Modelling Intercomparison Project (PLISMIP)—experimental design. Geosci. Model Dev..

[28] de Boer B (2015). Simulating the Antarctic ice sheet in the late-Pliocene warm period: PLISMIP-ANT, an ice-sheet model intercomparison project. Cryosphere.

[29] Sato T, Greve R (2012). Sensitivity experiments for the Antarctic ice sheet with varied sub-ice-shelf melting rates. Ann. Glaciol..

[30] Bernales J, Rogozhina I, Greve R, Thomas M (2016). Comparison of hybrid schemes for the combination of shallow approximations in numerical simulations of the Antarctic Ice Sheet. Cryosphere Discuss..

[31] Hindmarsh RCA (1999). On the numerical computation of temperature in an ice-sheet. J. Glaciol..

[32] Bragg FJ, Lunt DJ, Haywood AM (2012). Mid-Pliocene climate modelled using the UK Hadley Centre Model: PlioMIP Experiments 1 and 2. Geosci. Model Dev..

[33] Stepanek C, Lohmann G (2012). Modelling mid-Pliocene climate with COSMOS. Geosci. Model Dev..

[34] Rosenbloom NA, Otto-Bliesner BL, Brady EC, Lawrence PJ (2013). Simulating the mid-Pliocene Warm Period with the CCSM4 model. Geosci. Model Dev..

[35] Contoux C, Ramstein G, Jost A (2012). Modelling the mid-Pliocene Warm Period climate with the IPSL coupled model and its atmospheric component LMDZ5A. Geosci. Model Dev..

[36] Chan WL, Abe-Ouchi A, Ohgaito R (2011). Simulating the mid-Pliocene climate with the MIROC general circulation model: experimental design and initial results. Geosci. Model Dev..

[37] Kamae Y, Ueda H (2012). Mid-Pliocene global climate simulation with MRI-CGCM2.3: set-up and initial results of PlioMIP Experiments 1 and 2. Geosci. Model Dev..

[38] Zhang ZS (2012). Pre-industrial and mid-Pliocene simulations with NorESM-L. Geosci. Model Dev..

[39] Fretwell P (2013). Bedmap2: improved ice bed, surface and thickness datasets for Antarctica. Cryosphere.

[40] Dolan AM (2015). Using results from the PlioMIP ensemble to investigate the Greenland Ice Sheet during the mid-Pliocene Warm Period. Clim. Past.

[41] Koenig SJ (2015). Ice sheet model dependency of the simulated Greenland Ice Sheet in the mid-Pliocene. Clim. Past.

[42] Hill DJ (2014). Evaluating the dominant components of warming in Pliocene climate simulations. Clim. Past.

[43] Yan Q, Zhang Z, Wang H (2016). Investigating uncertainty in the simulation of the Antarctic ice sheet during the mid-Piacenzian. J. Geophys. Res.: Atmos..

[44] Austermann J (2015). The impact of dynamic topography change on Antarctic ice sheet stability during the mid-Pliocene warm period. Geology.

[45] Gasson E, DeConto R, Pollard D (2015). Antarctic bedrock topography uncertainty and ice sheet stability. Geophys. Res. Lett..

[46] Gasson E, DeConto R, Pollard D (2016). Modeling the oxygen isotope composition of the Antarctic ice sheet and its significance to Pliocene sea level. Geology.

[47] Moucha R (2008). Dynamic topography and long-term sea-level variations: there is no such thing as a stable continental platform. Earth Planet. Sci. Lett..

[48] Winnick MJ, Caves JK (2015). Oxygen isotope mass-balance constraints on Pliocene sea level and East Antarctic Ice Sheet stability. Geology.

[49] Raymo ME, Kozdon R, Evans D, Lisiecki L, Ford HL (2018). The accuracy of mid-Pliocene δ18O-based ice volume and sea level reconstructions. Earth Sci. Rev..

[50] Golledge NR (2015). The multi-millennial Antarctic commitment to future sea-level rise. Nature.

[51] Feldmann J, Albrecht T, Khroulev C, Pattyn F, Levermann A (2017). Resolution-dependent performance of grounding line motion in a shallow model compared with a full-Stokes model according to the MISMIP3d intercomparison. J. Glaciol..

[52] Cornford SL, Martin DF, Lee V, Payne AJ, Ng EG (2016). Adaptive mesh refinement versus subgrid friction interpolation in simulations of Antarctic ice dynamics. Ann. Glaciol..

[53] Dowsett H (2016). The PRISM4 (mid-Piacenzian) paleoenvironmental reconstruction. Clim. Past.

[54] Haywood AM (2016). The Pliocene Model Intercomparison Project (PlioMIP) Phase 2: scientific objectives and experimental design. Clim. Past.

[55] Salzmann U, Haywood AM, Lunt DJ, Valdes PJ, Hill DJ (2008). A new global biome reconstruction and data-model comparison for the Middle Pliocene. Glob. Ecol. Biogeogr..

[56] Hill, D. J. *Modelling Earth’s Cryosphere During Peak Pliocene Warmth*. Ph.D. Thesis, University of Bristol (2009).

[57] Hutter, K. *Theoretical Glaciology: Material Science of Ice and the Mechanics of Glaciers and Ice Sheets* (Springer, 1983).

[58] Morland, L. W. In *Dynamics of the West Antarctic Ice Sheet* (eds de Veen, C. J. V. & Oerlemans, J.) 99–116 (D. Reidel, 1987).

[59] Reerink TJ, Kliphuis MA, van de Wal RSW (2010). Mapping technique of climate fields between GCM’s and ice models. Geosci. Model Dev..

[60] Snyder, J. P. Map projections—a working manual. In *USGS Professional Paper 1395*. Available at: https://pubs.er.usgs.gov/publication/pp1395 (USGS, 1987).

[61] de Boer B, van de Wal RSW, Lourens LJ, Bintanja R, Reerink TJ (2013). A continuous simulation of global ice volume over the past 1 million years with 3-D ice-sheet models. Clim. Dyn..

[62] Winkelmann R (2011). The Potsdam Parallel Ice Sheet Model (PISM-PIK)—Part 1: model description. Cryosphere.

[63] Beckmann A, Goosse H (2003). A parameterization of ice shelf–ocean interaction for climate models. Ocean Model..

[64] Dunse T, Greve R, Schuler TV, Hagen JO (2011). Permanent fast flow versus cyclic surge behaviour: numerical simulations of the Austfonna ice cap, Svalbard. Ann. Glaciol..

[65] Marsiat I (1994). Simulation of the Northern Hemisphere continental ice sheets over the last glacial–interglacial cycle: experiments with a latitude-longitude vertically integrated ice sheet model coupled to a zonally averaged climate model. Paleoclimates.

[66] Reeh N (1991). Parameterization of melt rate and surface temperature on the Greenland ice sheet. Polarforschung.

[67] Calov R, Greve R (2005). A semi-analytical solution for the positive degree-day model with stochastic temperature variations. J. Glaciol..

[68] Holland PR, Jenkins A, Holland DM (2008). The response of ice shelf basal melting to variations in ocean temperature. J. Clim..

[69] Braithwaite RJ (1995). Positive degree-day factors for ablation on the Greenland ice sheet studied by energy-balance modelling. J. Glaciol..

[70] Hill DJ, Dolan AM, Haywood AM, Hunter SJ, Stoll DK (2010). Sensitivity of the Greenland Ice Sheet to Pliocene sea surface temperatures. Stratigraphy.

