# Supplementary material for: High climate model dependency of Pliocene Antarctic ice-sheet predictions
Source: Nat Commun. 2018 Jul 18;9:2799. doi: 10.1038/s41467-018-05179-4 (PMC6052068; doi:10.1038/s41467-018-05179-4)
Supplement: Supplementary file 1 — Supplementary Information [file 41467_2018_5179_MOESM1_ESM.pdf]

Supplementary Information

High Climate Model Dependency of Pliocene Antarctic Ice Sheet Predictions

Aisling M. Dolan, Bas de Boer, Jorge Bernales, Daniel J. Hill and Alan M. Haywood

Supplementary Note 1: Comparison of control simulations of the modern Antarctic Ice-Sheet to Observations

There are a number of ways in which ice-sheet predictions from our control simulations could be assessed in order to ensure that a certain combination of General Circulation Model (GCM) and Ice Sheet Model (ISM) produce a sensible representation of the modern-day Antarctic Ice Sheet (AIS). Here we have compared the predictions of volume, area and thickness with the Bedmap2 reconstruction[1], but also considered whether a particular GCM-ISM predicts a volume that falls outside of the ensemble mean (± one standard deviation – see Methods; Supplementary Table 2 and 3).

Supplementary Fig. 7 compares predicted East Antarctic Ice Sheet (EAIS) volume and area for ANICE and SICOPOLIS ISMs in the control scenarios. Although all of the model combinations do a reasonable job of simulating the EAIS area (compared to data), the MRI-CGCM2.3 clearly overestimates the volume of the EAIS relative to the other model combinations and the Bedmap2 reconstruction. For West Antarctica, the simulations using MIROC4m stand out as being significantly lower than the other PlioMIP models when using ANICE and SICOPOLIS, both in terms of volume and area (Supplementary Fig. 8). The predictions using MIROC4m also are lower than the volume and area derived from Bedmap2. West Antarctic Ice Sheet (WAIS) volume predictions using MRI-CGCM2.3 and ANICE are also clearly too large in comparison to modern, however the predictions using SICOPOLIS do not stand out significantly from the other model combinations.

The Root-mean square error (RMSE) in terms of predicted ice sheet thickness for EAIS and WAIS shows that MRI-CGCM2.3 is over predicting ice sheet thickness for East Antarctica, which is consistent with the high EAIS volumes predicted for this model (Supplementary Fig. 9). Both ISMs show high errors in predicting the land ice for West Antarctica using the MIROC4m climate model, which is reflected in the collapse of the WAIS in the control simulations (see also Fig. 2). For MRI-CGCM2.3, SICOPOLIS predicts a reasonable ice sheet thickness, but the ANICE ISM predicts a WAIS and associated ice shelves that are too thick when compared to Bedmap2.

Using the performance criteria outlined in the Methods section of the main paper (Supplementary Tables 2 and S3) and the comparison of predicted modern volume, area and ice-sheet thickness, two GCM-ISM combinations consistently perform poorly against the metrics we have chosen (MRI-CGCM2.3 and MIROC4m). Therefore, these models have been excluded from the Pliocene Scenarios presented in this study.

Supplementary Note 2: Understanding the reasons that certain GCM-ISM combinations lead to a poor representation of the modern Antarctic Ice Sheet

In all of the ISMs, the MRI-CGCM2.3 climate forcing results in an unrealistically large AIS, which is extensively thicker than the Bedmap2 reconstruction, especially over areas of continental East Antarctica (Fig. 2; Supplementary Fig. 7). Annual average Antarctic precipitation in the MRI-CGCM2.3 is almost double that of other models for the pre-industrial control (0.8 mm day^{-1}) and it is the coldest model with an annual average AIS temperature of -38.3°C (Fig. 1). Combined, these factors contribute to the most positive surface mass balance (SMB) predictions over both East and West Antarctica when forcing the ISMs (approximately 2000 Gt yr^{-1}; Supplementary Fig. 4). When compared to observations of climate, it has been shown that MRI-CGCM2.3 exhibits a strong Austral summer cold bias over Antarctica (of over 8°C), which is attributed to cloud concentration reduction in the model (when compared with a previous version of the model – MRI-CGCM2.0)[2]. MRI-CGCM2.3 also has very different snow albedo values (α = up to 0.9) to the other PlioMIP models (α = 0.55-0.7), which may also contribute to the colder temperatures allowing for a larger modern-day AIS to be predicted. MRI-CGCM2.3 largely overestimates precipitation in comparison to observations when compared zonally between 70°S and 90°S (and this is strongest in the Austral summer months)[2]. We suggest therefore, that the regional model biases exhibited by MRI-CGCM2.3 are likely to be partly the cause of such large volumetric predictions for AIS when using all three ISMs.

Present-day AIS reconstructions using MIROC4m also do not give a reasonable representation of the modern ice-sheet. The simulations using ANICE and SICOPOLIS both exhibit a low volume associated with the collapse of the WAIS in both ISMs. In part, this may be driven by the fact that MIROC4m exhibits the second highest temperatures and lowest precipitation rates over West Antarctica of all of the PlioMIP models (Supplementary Fig. 1). Additionally, the removal of ice in this area is likely a response to warm sub-shelf temperatures (up to 1.6°C) in the Marie Byrd area of the Ross Ice Shelf, which are associated with a negative basal mass balance (Supplementary Fig. 4). On average, the area of the Ross and Ronne Ice shelves is greater in the simulations using ANICE, than those using SICOPOLIS, with only the simulation using the MIROC4m forcing showing a loss of both ice-shelves for present day (Fig 2). This difference could be attributed to the different exposed shelf melt rates prescribed in the models for ANICE and SICOPOLIS, and the applied calving rate in SICOPOLIS (see Methods). In the SICOPOLIS model, this is also a result of a negative SMB on land, leading to an insufficient ice flux from the ice-sheet to sustain the ice-shelves.

The performance of MIROC4m over the Antarctic region has not been addressed in previous model description papers[3], however, we note that the different treatment of the land-sea mask in MIROC4m relative to the other PlioMIP models (even for the control simulation) may be one reason that the sub-shelf temperatures are particularly high in this model. MIROC4m treats the ice-shelves as ocean grid points allowing sea-ice to grow over them (rather than as land in the other models).

Supplementary Figures

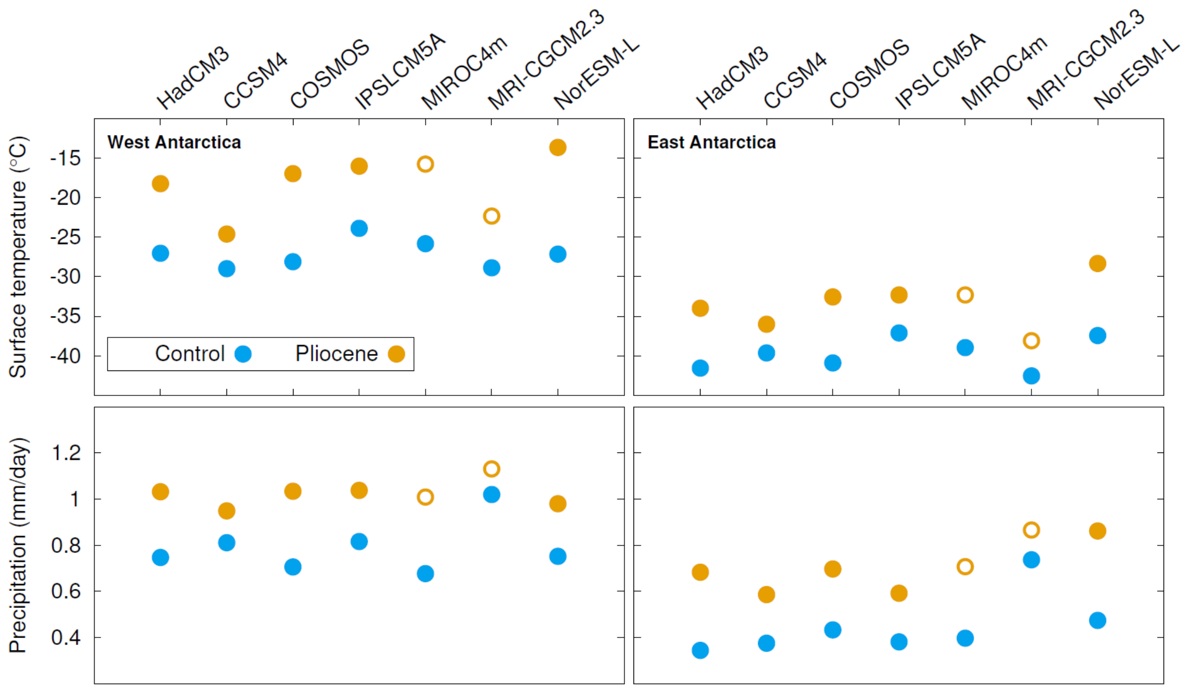

Supplementary Figure 1. Pliocene climatological averages over West and East Antarctica. Average mean annual surface air temperature (°C) and precipitation (mm day^{-1}) over West and East Antarctica as predicted by the PlioMIP models for the Control (pre-industrial) and Pliocene climate (see Haywood et al., 2013; Supplementary Table 1). Unfilled circles (for MIROC4m and MRI-CGCM2.3) denote climatologies for the Pliocene that were not used to force ISMs in this study.

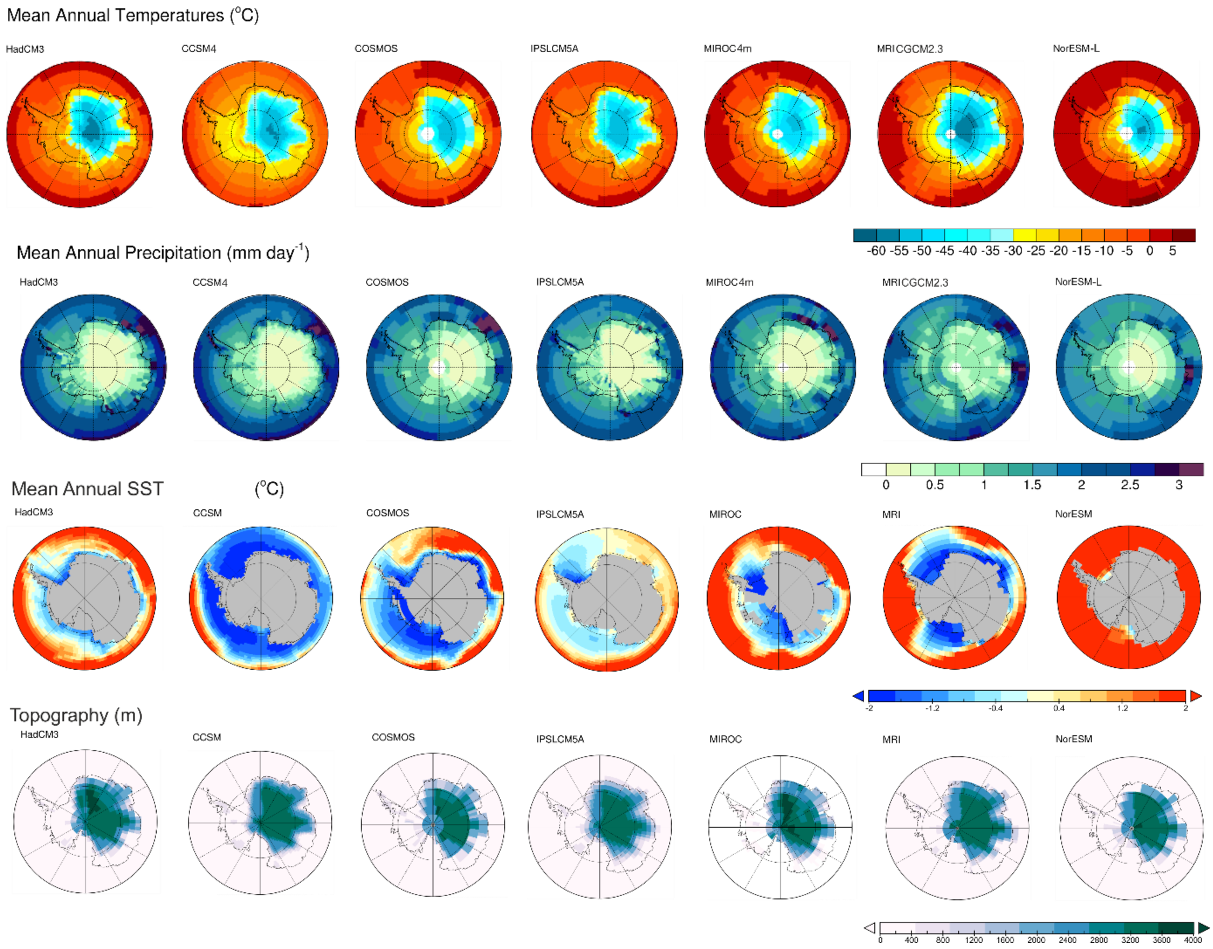

Supplementary Figure 2. Pliocene climatological forcing from the seven PlioMIP climate models. Pliocene mean annual surface air temperature (°C), precipitation (mm day^{-1}) and sea surface temperature (°C) predicted by the PlioMIP models (see Haywood et al., 2013; Table 1). The bottom row shows Antarctic topography (m) from each climate model.

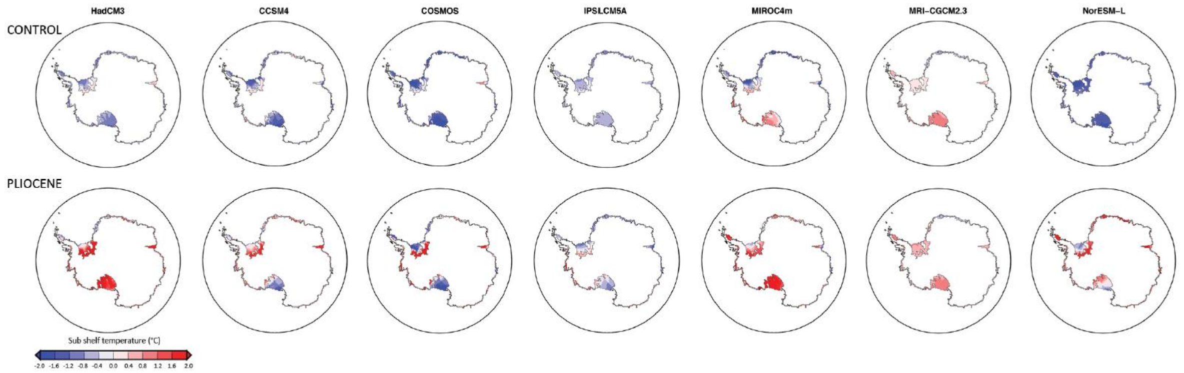

Supplementary Figure 3. Modern and Pliocene sub-shelf temperatures. Pre-Industrial (Modern) and Pliocene mean annual sub-shelf temperature (°C) as predicted by the PlioMIP climate models. Note that the sub-shelf temperatures have been extrapolated from the nearest ocean grid point in the GCM (due to the nature of the land-sea mask in the GCMs – see Methods and Supplementary Fig 2).

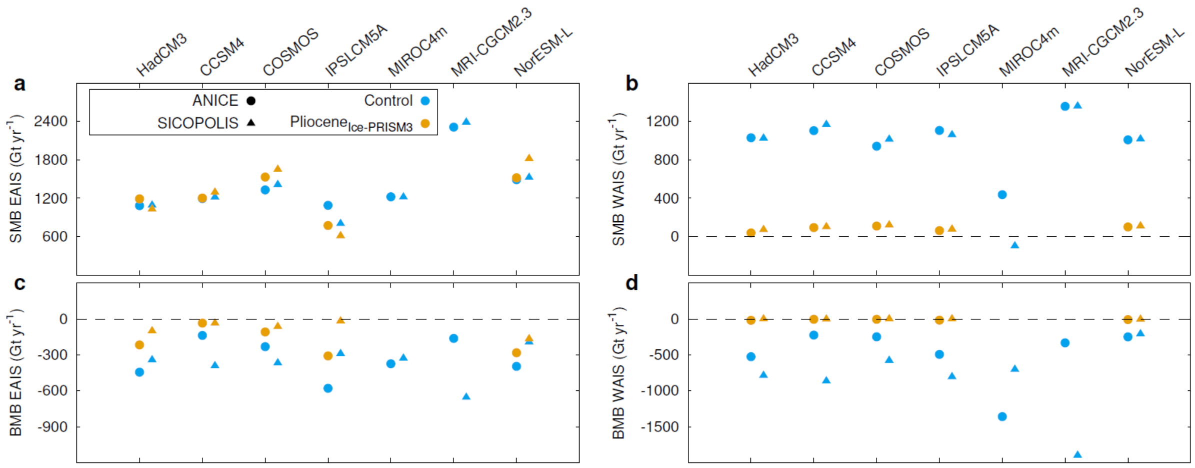

Supplementary Figure 4. Surface and Basal Mass Balance. Pliocene surface and basal mass balance (Gt yr^{-1}) predictions over East and West Antarctica. Mass balance is given as an average of all time-steps within the ISM simulation.

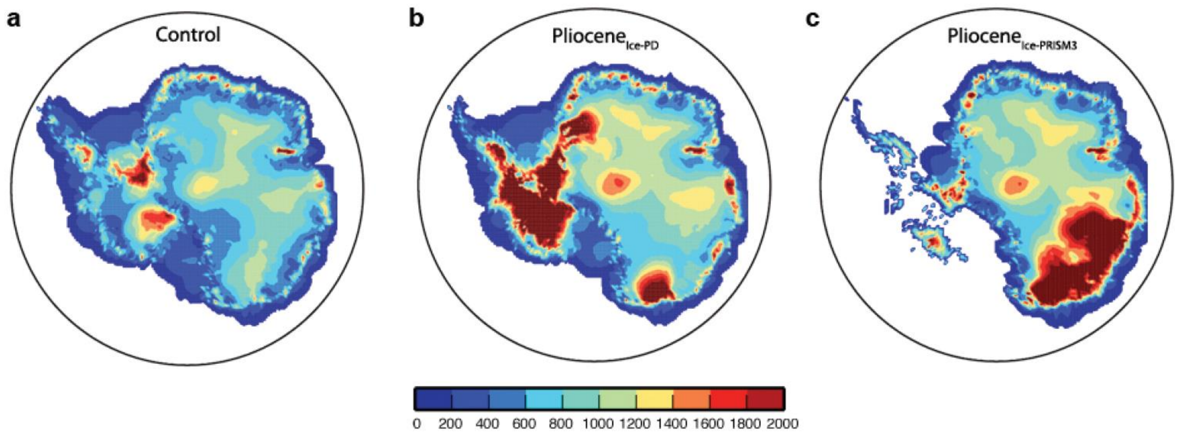

Supplementary Figure 5. Ice-Sheet Thickness Range. The range in ice-sheet thicknesses (m) predicted for the Control, Pliocene$_{Ice-PD}$ and Pliocene$_{Ice-PRISM3}$ simulations from all of the climate models.

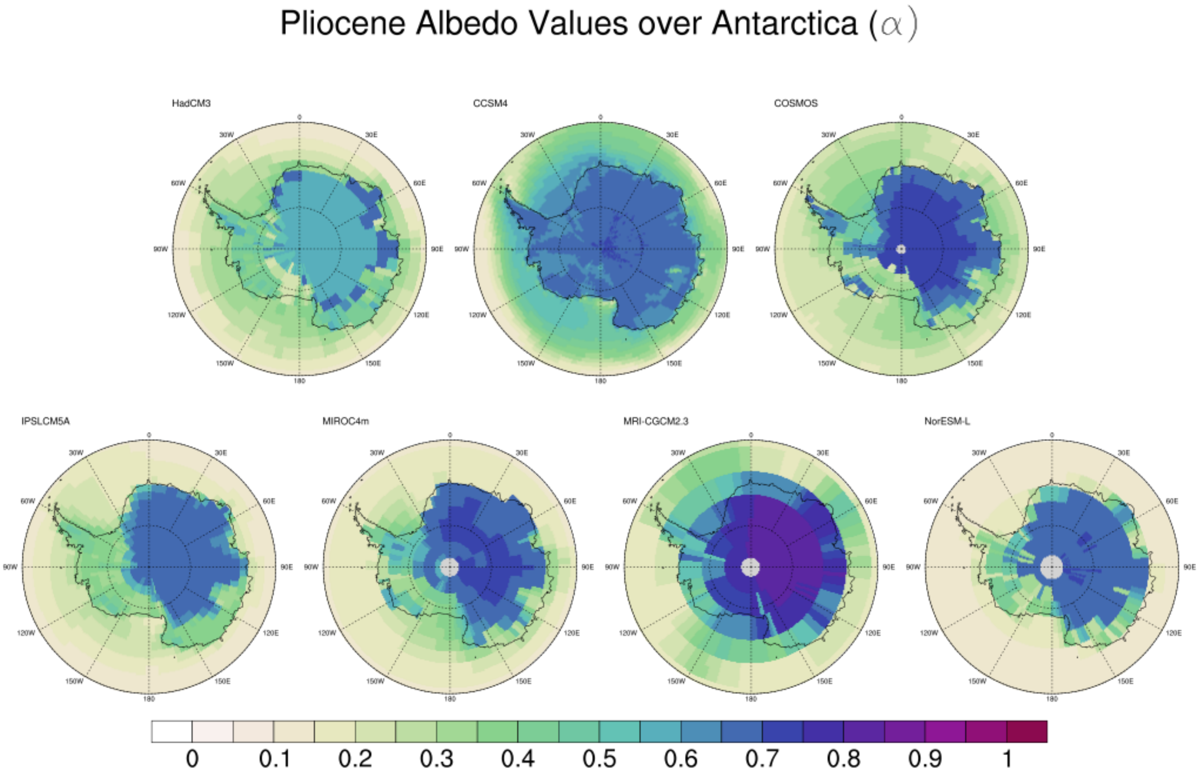

Supplementary Figure 6. Pliocene Albedo Values. Annual mean clear sky albedo values over Antarctica for the PlioMIP climate models.

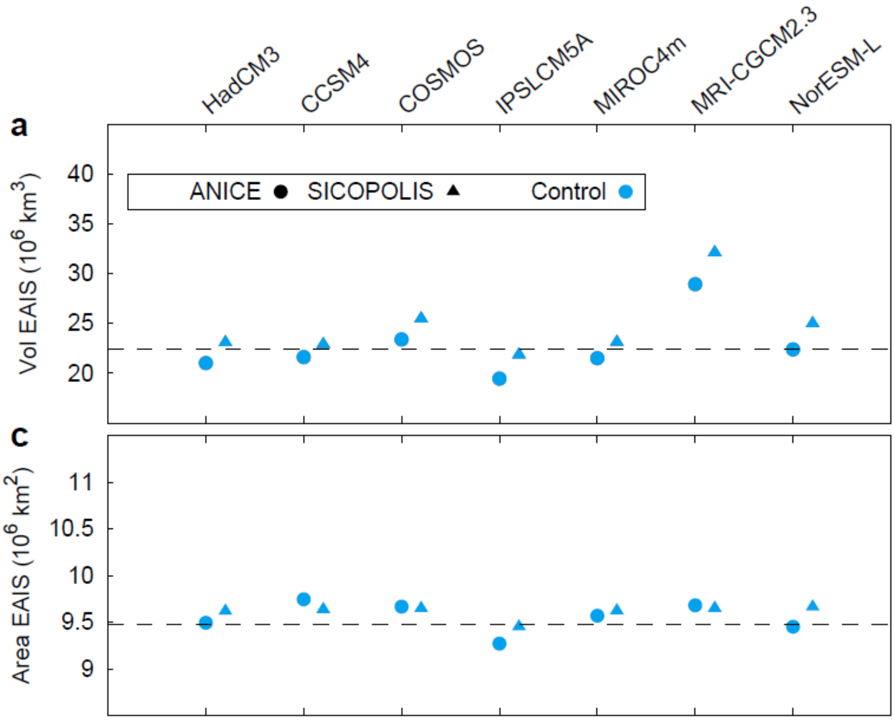

Supplementary Figure 7. Simulated Modern East Antarctic Ice Sheet Volume and Area. Comparison of predicted volume (top) and area (bottom) for East Antarctica from the two SSA-SIA ISMs (ANICE and SICOPOLIS) for each of the PlioMIP models. Horizontal line denotes the modern day volume and area as derived from the Bedmap2 reconstruction.

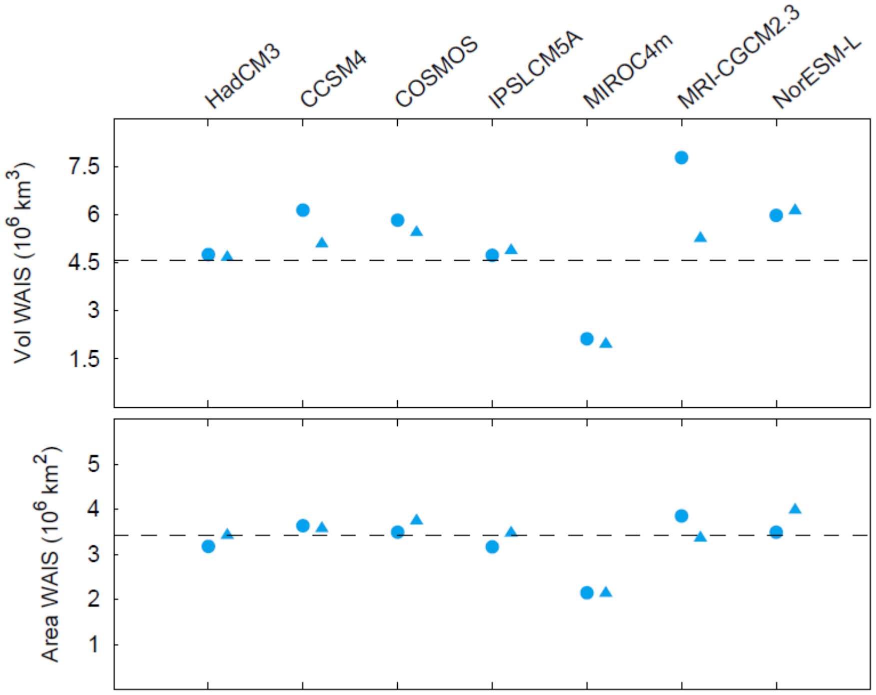

Supplementary Figure 8. Simulated Modern West Antarctic Ice Sheet Volume and Area. Comparison of predicted volume (top) and area (bottom) for West Antarctica from the two SSA-SIA ISMs (ANICE and SICOPOLIS) for each of the PlioMIP models. Horizontal line denotes the modern day volume and area as derived from the Bedmap2 reconstruction.

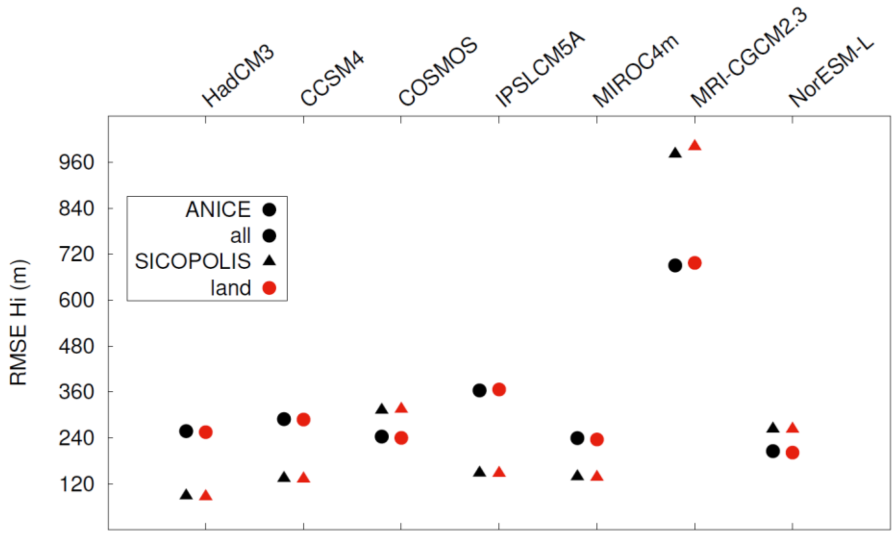

Supplementary Figure 9. Simulated Error in EAIS Thickness. RMSE of ice sheet thickness for East Antarctica from the two SSA-SIA ISMs (ANICE and SICOPOLIS) for each of the PlioMIP climate models when compared with the Bedmap2 reconstruction. ‘All’ refers to the combined errors when considering land ice and also ice shelves.

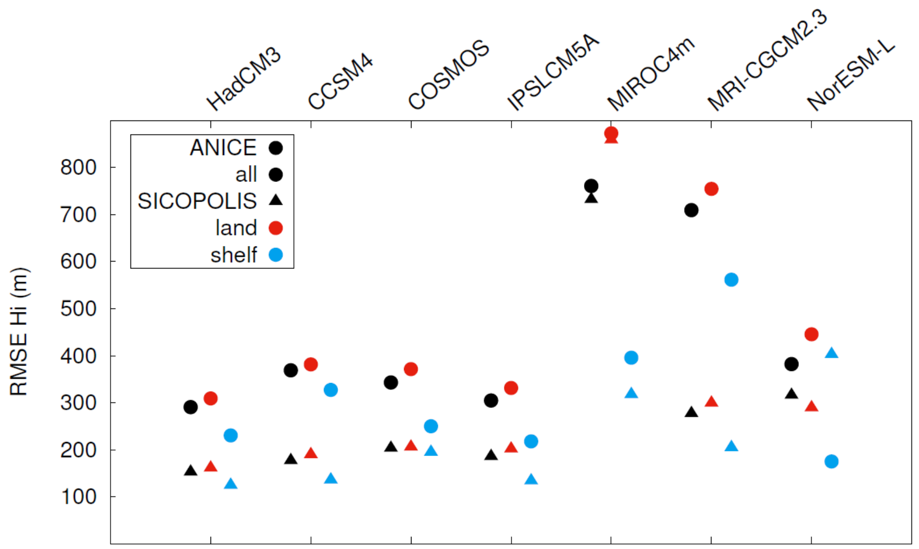

Supplementary Figure 10. Simulated Error in WAIS Thickness. RMSE of ice sheet thickness for West Antarctica and the ice shelves from the two SSA-SIA ISMs (ANICE and SICOPOLIS) for each of the PlioMIP climate models when compared with the Bedmap2 reconstruction. 'All' refers to the combined errors when considering land ice and also ice shelves. For West Antarctica, we have shown the ice shelf RMSE separately.

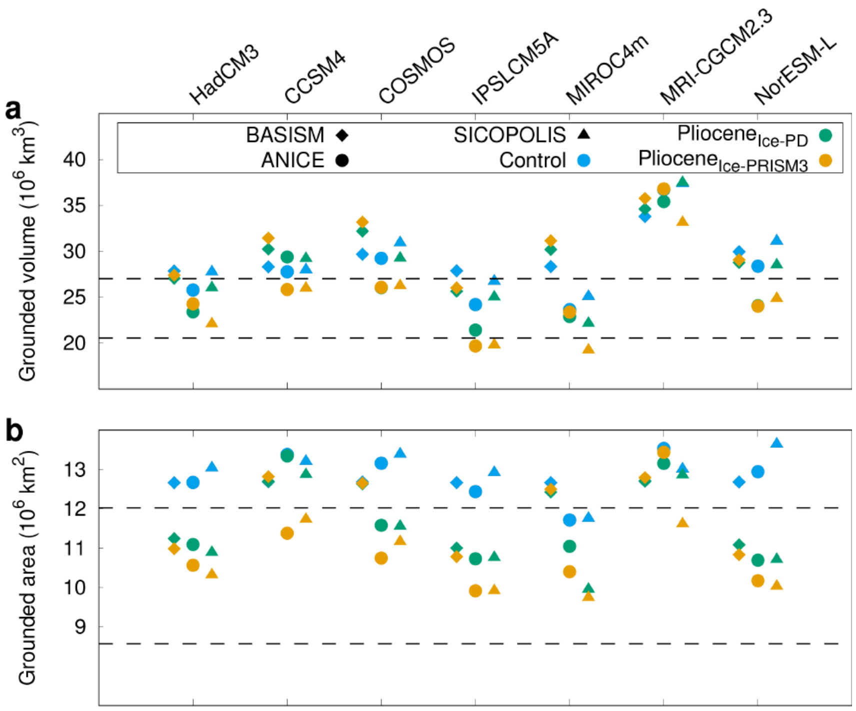

Supplementary Figure 11. Simulated ice-sheet volume and area. Grounded ice-sheet volume (× 10^6 km^3) and area (× 10^6 km^2) prediction for each ISM given each of the climate model forcings for the Control, Pliocene$_{Ice-PD}$ and Pliocene$_{Ice-PRISM3}$ experiments. Horizontal dashed lines show the PRISM3 ice-sheet volume/area[4] (bottom dashed lines) and the Bedmap2[1] modern volume/area (top dashed lines). This figure is the same as Figure 4 in the main text, however it includes the results from the two GCM-ISM combinations that were not included in the main Pliocene analysis (MIROC4m and MRI-CGCM2.3). This has not been presented in the main text as when used to force the ISMs in this study, the GCM-ISM combinations do not produce a reasonable representation of the present-day AIS (see Supplementary Note 1).

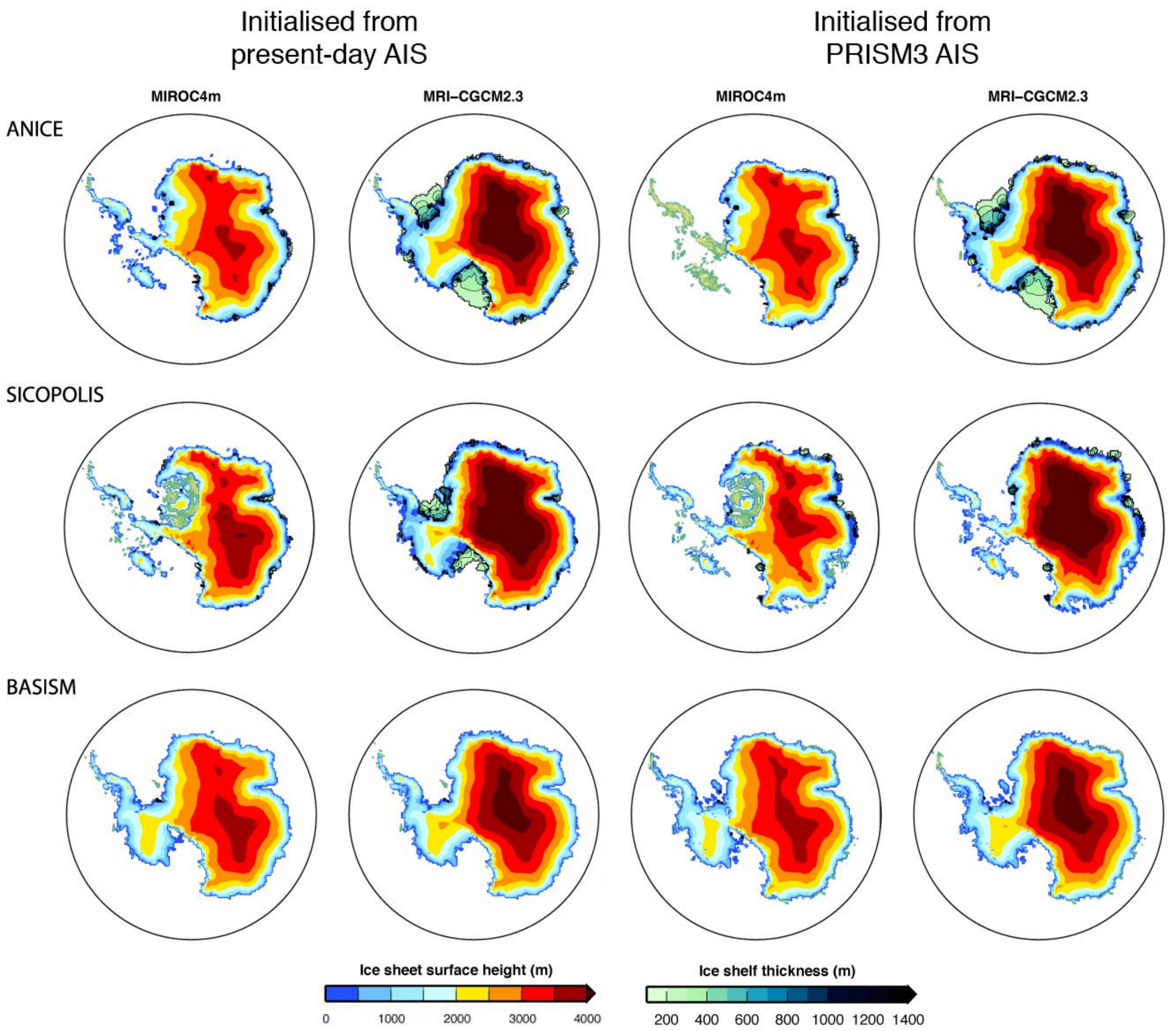

Supplementary Figure 12. Simulated Pliocene ice-sheets by MIROC4m and MRI-CGCM2.3. ISM predictions of grounded ice-sheet surface height (m) and ice-shelf thickness (m) for the Pliocene$_{Ice\text{-}PRISM3}$ and the Pliocene$_{Ice\text{-}PD}$ experiments using the climatological forcing fields from the PlioMIP climate models MIROC4m and MRI-CGCM2.3. Note that the ice-shelves are only simulated by the ANICE and SICOPOLIS ISMs. These ice-sheet reconstructions have not been presented in the main text as when used to force the ISMs in this study, the GCM-ISM combinations do not produce a reasonable representation of the present-day AIS (see Supplementary Note 1).

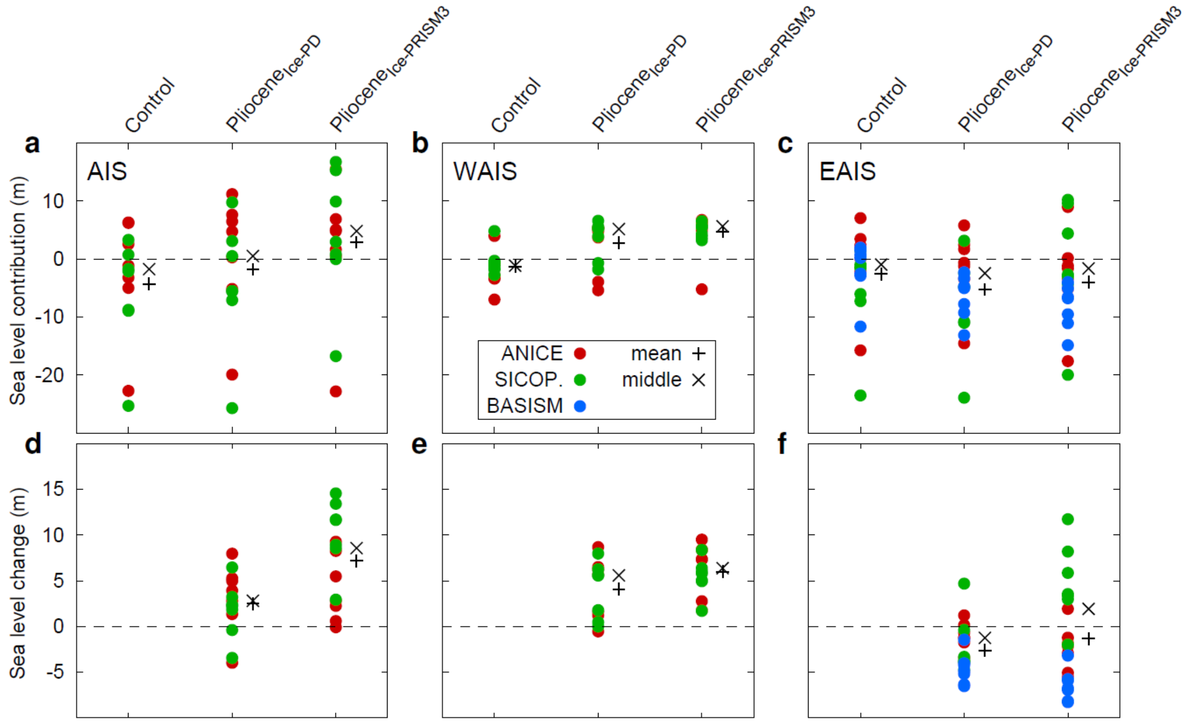

Supplementary Figure 13. Sea level contributions. Sea level contribution relative to Bedmap2 of the total ice volume of the a) AIS, b) WAIS and c) EAIS. The relative change in sea level for the two Pliocene experiments (Pliocene – Control experiment shown in panels a-c) for d) AIS, e) WAIS and f) EAIS. Since BASISM is a SIA-only model, results are only shown for the EAIS. This figure is the same as Figure 7 in the main text, however it includes the sea level contributions from the two GCM-ISM combinations that were not included in the main Pliocene analysis (MIROC4m and MRI-CGCM2.3). As such the middle is the 7th ranking sea level contribution from the list of 14 SIA-SSA model results.

Supplementary Tables

Supplementary Table 1. Details of the PlioMIP climate models used to force the ice-sheet models. This includes the resolution of the atmosphere components and ocean components of the models and the main reference for each model. The Land-Sea Mask (LSM) scheme implemented by each model is also detailed (see Haywood et al., 2010). Regarding the LSM, "preferred" refers to a LSM that has been entirely altered to meet the PlioMIP boundary conditions (e.g. the creation of a West Antarctic seaway within the models). "Alternate" is where modelling groups have had to use a more similar to modern LSM. More comprehensive details of each model, and their implementation of the LSM, can be found in Haywood et al. (2013) and the individual references listed in this table.

Model Name (key reference)	Atmosphere Resolution (Latitude × Longitude)	Ocean Resolution (Latitude × Longitude)	Preferred or Alternate LSM
CCSM4[5]	0.90° × 1.25°	1.0° × 1.0°	Alternate
COSMOS[6]	3.75° x 3.75°	3.0° × 1.8°	Preferred
HadCM3[7]	2.50° x 3.75°	1.25° × 1.25°	Alternate
IPSLCM5A[8]	1.90° x 3.75°	0.5°-2.0° × 2.0°	Alternate
MIROC4m[9]	~ 2.80° × 2.80° (T42)	0.5°-1.4° × 1.4°	Preferred
MRI-CGCM2.3[10]	~ 2.80° × 2.80° (T42)	0.5°-2.0° × 2.5°	Alternate
NorESM-L[11]	~ 3.75° × 3.75° (T31)	~ 3.0° × 3.0°	Alternate

Supplementary Table 2. Predicted modern Antarctic Ice-Sheet volumes. The predicted modern Antarctic ice-sheet volume (× 10^6 km^3) of each ISM simulation driven by the PlioMIP GCM forcing climates.

GCM	ANICE	SICOPOLIS	BASISM
CCMS4	27.762	27.964	28.302
COSMOS	29.224	30.902	29.670
HadCM3	25.779	27.733	27.849
IPSLCM5A	24.193	26.716	27.868
MIROC4m	23.638	25.065	28.338
MRI-CGCM2.3	36.720	37.363	33.786
NorESM-L	28.376	31.098	29.945

Supplementary Table 3. Evaluation of predicted modern Antarctic Ice-Sheet volumes. The mean volume (× 10^6 km^3) and standard deviation in predicted ice-sheet volume within the ensemble of control results removing one GCM at a time (i.e. the mean of the other 18 ensemble members). Also shown is whether or not the predicted volumes (from at least 2 of the three ISM simulations) forced with the climate from the removed model falls within the range (mean ± 1 standard deviation). Where the GCM does not fall within the identified range it has been removed from the Pliocene assessment (e.g. MIROC4m and MRI-CGCM2.3)

Removed GCM	CCSM4	COSMOS	HadCM3	IPSLCM5A	MIROC4m	MRI-CGCM2.3	NorESM-L
Mean	29.13	28.81	29.27	29.42	29.51	27.80	28.83
Standard Deviation (SD)	3.83	3.82	3.74	3.60	3.46	2.09	3.81
Mean +SD	32.96	32.63	33.02	33.02	32.97	29.90	32.64
Mean -SD	25.29	24.98	25.53	25.82	26.05	25.71	25.02
Removed model fall within range	YES	YES	YES	YES	NO	NO	YES

Supplementary Table 4. Physical parameters for the sub-shelf melt rate parameterization.

Constant and description		Value
ρ_{i}	Ice density (kg m^{-3})	910
ρ_{w}	Seawater density (kg m^{-3})	1028
$\mathrm{c}p_{\mathrm{O}}$	Specific heat capacity of ocean (J kg^{-1} $°C^{-1}$)	3974
γT	Thermal exchange velocity (m s^{-1})	10^{-4}
L	Latent heat of fusion (J kg^{-1})	3.35×10^5
S_{O}	Mean salinity of the ocean (psu)	35

Supplementary Information References

1. Fretwell P*, et al.* Bedmap2: improved ice bed, surface and thickness datasets for Antarctica. *The Cryosphere* **7**, 375-393 (2013).

2. Yukimoto S*, et al.* Present-Day Climate and Climate Sensitivity in the Meteorological Research Institute Coupled GCM Version 2.3 (MRI-CGCM2.3). *Journal of the Meteorological Society of Japan* **84**, 333-363 (2006).

3. Developers K-M. K-1 Coupled Model (MIROC) Description: K1 Technical Report 1. (ed^(eds Hasumi H, Emori S). University of Tokyo (2004).

4. Dowsett HJ*, et al.* The PRISM3D paleoenvironmental reconstruction. *Stratigraphy* **7**, 123-139 (2010).

5. Rosenbloom NA, Otto-Bliesner BL, Brady EC, Lawrence PJ. Simulating the mid-Pliocene Warm Period with the CCSM4 model. *Geosci Model Dev* **6**, 549-561 (2013).

6. Stepanek C, Lohmann G. Modelling mid-Pliocene climate with COSMOS. *Geosci Model Dev* **5**, 1221-1243 (2012).

7. Bragg FJ, Lunt DJ, Haywood AM. Mid-Pliocene climate modelled using the UK Hadley Centre Model: PlioMIP Experiments 1 and 2. *Geosci Model Dev* **5**, 1109-1125 (2012).

8. Contoux C, Ramstein G, Jost A. Modelling the mid-Pliocene Warm Period climate with the IPSL coupled model and its atmospheric component LMDZ5A. *Geosci Model Dev* **5**, 903-917 (2012).

9. Chan WL, Abe-Ouchi A, Ohgaito R. Simulating the mid-Pliocene climate with the MIROC general circulation model: experimental design and initial results. *Geosci Model Dev* **4**, 1035-1049 (2011).

10. Kamae Y, Ueda H. Mid-Pliocene global climate simulation with MRI-CGCM2.3: set-up and initial results of PlioMIP Experiments 1 and 2. *Geosci Model Dev* **5**, 793-808 (2012).

11. Zhang Z, Yan Q. Pre-industrial and mid-Pliocene simulations with NorESM-L: AGCM simulations. *Geosci Model Dev* **5**, 1033-1043 (2012).
